# ENO1-Related Signaling in Hypertension-Associated Atrial Remodeling: Evidence Implicating PI3K/AKT and NOX4

**DOI:** 10.3390/cells15211970

**Published:** 2026-10-29

**Authors:** Yang Lou, Qian Zhu, Yuhang Tao, Yameng Chen, Shiquan Chen, Zuwen Zhang, Hongqiang Cheng, Guosheng Fu, Chenyang Jiang

**Affiliations:** 1 Department of Cardiology, Sir Run Run Shaw Hospital, Zhejiang University School of Medicine, Hangzhou 310016, China; louy9602@zju.edu.cn (Y.L.); zhuqian427@zju.edu.cn (Q.Z.); taoyuhang@zju.edu.cn (Y.T.); chen12012426@163.com (Y.C.); 3412140@zju.edu.cn (S.C.); zzw0715@163.com (Z.Z.); hqcheng11@zju.edu.cn (H.C.); 2 Center for Cardiac Arrhythmias and Cardiomyopathy (Clinical & Research), Sir Run Run Shaw Hospital, Zhejiang University School of Medicine, Hangzhou 310016, China; 3 Zhejiang Key Laboratory of Cardiovascular Intervention and Precision Medicine, Hangzhou 310016, China; 4 Engineering Research Center for Cardiovascular Innovative Devices of Zhejiang Province, Hangzhou 310016, China

**Keywords:** hypertension, ENO1, atrial fibrillation, oxidative stress, NOX4

## Abstract

**Highlights:**

**What are the main findings?**

- Multi-omics analyses identified persistent atrial ENO1 upregulation in hypertensive rats, with concordant increases in two human atrial fibrillation datasets.
- ENO1 knockdown attenuated Ang II-induced hypertrophic and oxidative-stress responses, whereas PI3K activation increased PI3K/AKT phosphorylation and NOX4 expression under ENO1-silenced conditions. In vivo, AP-III-a4 treatment attenuated atrial remodeling and AERP shortening and significantly shortened pacing-induced AF duration; AF inducibility was numerically lower but not statistically significant.

**What are the implications of the main findings?**

- These findings suggest that PI3K/AKT signaling may be involved in ENO1-associated regulation of NOX4 expression.
- ENO1-related signaling may represent a potential new direction for developing future therapeutic strategies for AF.

**Abstract:**

Atrial fibrillation (AF) is the most prevalent sustained cardiac arrhythmia worldwide; hypertension is one of the most important risk factors for AF and contributes to both its initiation and maintenance. This study investigated the contribution of enolase 1 (ENO1) to hypertension-associated atrial remodeling and susceptibility to pacing-induced AF. Long-term hypertension was associated with increased AF inducibility in rats, and atrial transcriptomic analyses at two stages of hypertension identified persistent ENO1 upregulation. ENO1 was also increased in two external human left atrial AF datasets. An angiotensin II (Ang II)-induced atrial cardiomyocyte injury model was constructed to evaluate the role of ENO1 in Ang II-induced cardiomyocyte hypertrophy. In Ang II-stimulated primary neonatal rat atrial cardiomyocytes, siRNA-mediated ENO1 knockdown attenuated hypertrophic and oxidative-stress-related changes and was accompanied by lower PI3K/AKT phosphorylation and NADPH oxidase 4 (NOX4) expression. AP-III-a4 treatment produced concordant changes in selected cellular endpoints. PI3K inhibition reduced NOX4 expression and intracellular hydrogen peroxide levels. In ENO1-silenced cardiomyocytes, PI3K activation with 740Y-P partially increased both PI3K/AKT phosphorylation and NOX4 expression. In Ang II-infused hypertensive mice, AP-III-a4 treatment reduced atrial enlargement, fibrosis, PI3K/AKT phosphorylation, NOX4 expression, and atrial reactive oxygen species signals; it also attenuated atrial effective refractory period shortening. AP-III-a4 significantly shortened the duration of pacing-induced AF and was associated with numerically lower AF inducibility, although the latter difference did not reach statistical significance. Collectively, these findings support the involvement of PI3K/AKT signaling in ENO1-associated regulation of NOX4 and suggest that this relationship may contribute to the observed attenuation of hypertension-associated atrial remodeling.

## 1. Introduction

Atrial fibrillation (AF) is one of the most common cardiac arrhythmias worldwide, affecting 1–2% of the general population and up to 15% of those aged 80 or over [1]. According to the Global Burden of Cardiovascular Diseases report, nearly 60 million people had AF in 2023 [2], and there are approximately 5 million new cases annually [3]. With the increasing elderly population, the prevalence of AF is also expected to increase further over the next decade. Abnormal atrial hemodynamics in patients with AF predispose them to thromboembolic events and are associated with an increased risk of stroke, heart failure, and dementia [4]. Although catheter ablation techniques, including radiofrequency ablation [5], cryoballoon ablation [6], and pulsed-field ablation, can reduce the AF burden [7], the recurrence rate of AF after catheter ablation remains as high as 20–30% [8]. Most current therapies are symptomatic or palliative and have limited effects on incident AF. Elucidating the mechanisms underlying AF is therefore essential for prevention and for reducing its medical and socioeconomic burden.

A number of diseases and adverse lifestyle factors increase one’s susceptibility to AF. Early epidemiological studies showed that age or conditions such as heart failure, myocardial infarction, diabetes, and hypertension were risk factors [9]. Hypertension, also known as high blood pressure, is among the most prevalent cardiovascular diseases worldwide and causes multi-organ damage [10]. AF is an important cardiac manifestation of hypertension-mediated organ damage [11]. Patients with hypertension or those receiving antihypertensive therapy have been reported to have a 73% higher likelihood of AF than normotensive individuals [12,13] and 70–90% of patients with AF have hypertension [3]. However, the mechanisms by which hypertension promotes AF remain incompletely understood. Defining these mechanisms may improve our understanding of AF and provide new directions and guidance for pharmacological treatment.

Mitochondria are the principal source of energy in atrial cardiomyocytes. Mitochondrial dysfunction, including that associated with hypertension, can increase intracellular oxidative stress and contribute to susceptibility to AF [14,15,16]. Alpha-enolase (ENO1) is a central glycolytic enzyme that catalyzes the conversion of 2-phosphoglycerate to phosphoenolpyruvate and directly affects cellular energy production and metabolic rate. In addition to glycolysis, ENO1 has multiple nonenzymatic roles in tumors, immunity, and signal transduction. Recent studies have reported increased ENO1 expression in the brains of animals with hypertension [17]. Other studies suggest that ENO1 inhibition can attenuate pathological ventricular hypertrophy [18,19] and modulate the phenotypic switching of vascular smooth muscle cells [20,21]. However, the role of ENO1 in hypertensive AF and atrial oxidative stress remains unclear.

In this study, integrated transcriptomic analyses of atrial tissue at different stages of hypertension identified ENO1 as a persistently upregulated candidate molecule. Complementary Ang II-based cellular and mouse models, together with transcriptomic and proteomic analyses, were used to investigate the contribution of ENO1-related signaling to hypertension-associated atrial remodeling and susceptibility to pacing-induced AF.

## 2. Materials and Methods

### 2.1. Materials

Ang II (HY-13948), AP-III-a4 (HY-15858), LY294002 (HY-10108) and 740Y-P (HY-P0175) were purchased from MedChemExpress (Monmouth Junction, NJ, USA). Primary antibodies against ANP (27427-1-AP), β-actin (66009-1-Ig), MYH7 (22280-1-AP), cTnI (26152-1-AP), and NOX4 (14347-1-AP) were obtained from Proteintech (Wuhan, China); ENO1 antibody (ab227978) was obtained from Abcam (Cambridge, UK). Antibodies against AKT (#2972), p-AKT (#9271), PI3K (#4257), and p-PI3K (#17366) were purchased from Cell Signaling Technology (Danvers, MA, USA). The HRP-conjugated secondary antibodies AffiniPure Goat Anti-Mouse IgG (FDM007) and HRP AffiniPure Goat Anti-Rabbit IgG (FDR007) were obtained from FUDE Biological Technology (Hangzhou, China). Alexa Fluor 488 Goat Anti-Rabbit IgG (A-11034) and Alexa Fluor 555 Goat Anti-Mouse IgG (A-21422) antibodies were obtained from Thermo Fisher Scientific (Waltham, MA, USA). The JC-1 kit (C2005), DHE superoxide assay kit (S00064S), H_2_O_2_ kit (S0038), and Hoechst (C1011) stain and BrdU (SH-0136) were obtained from Beyotime (Shanghai, China). MitoSOX (M36008) and MitoTracker (M7512) were purchased from Thermo Fisher Scientific (Waltham, MA, USA). Collagenase type II (LS001476) was obtained from Worthington Biochemical Corporation (Lakewood, NJ, USA). DMEM, Opti-MEM, Lipofectamine RNAiMAX was purchased from Thermo Fisher Scientific (Waltham, MA, USA), D-HBSS from GENOM BIO (Hangzhou, China). DMSO, RIPA buffer, PMSF, trypsin digestion solution, and related reagents were obtained from Solarbio (Beijing, China).

### 2.2. Animals and Experimental Protocols

All animal experiments were performed in accordance with the regulations for laboratory animal management of Zhejiang University. Male 12-week-old spontaneously hypertensive rats (SHRs) and Wistar–Kyoto (WKY) rats, weighing 180–200 g, were obtained from the Vital River Laboratory Animal Technology Co., Ltd. (Beijing, China). The animals were housed under specific-pathogen-free (SPF), temperature-controlled conditions with a 12 h light/dark cycle and free access to food and water.

Male C57BL/6J mice aged 7 weeks and weighing 21–24 g were purchased from the Laboratory Animal Center, Hangzhou Medical College (Hangzhou, China). The mice were housed under the same conditions as the rats.

The animal study protocol was approved by the Ethical Review Committee and Laboratory Animal Welfare Committee of the Sir Run Run Shaw Hospital (NO. SRRSH2025-0063, approval date: 11 March 2025). All animal experiments were conducted in accordance with the approved institutional protocol and were reported in accordance with the ARRIVE 2.0 guidelines.

After one week of acclimation, hypertension was induced in the mice by Ang II infusion for 21 days via subcutaneous osmotic mini-pumps at 2000 ng/kg/min [22,23,24,25]. The Ang II solution was prepared in saline according to body weight and loaded into osmotic mini-pumps (1004W, RWD Life Science, Shenzhen, China); control mice received equal volumes of saline. The surgical procedure was performed as previously described [22,23]. Briefly, mice were anesthetized with inhaled isoflurane, a small incision was made beneath the right scapula, a subcutaneous pocket was created, and the pump was implanted. The incision was sutured and disinfected. No formal prospective sample-size calculation was performed. Animal numbers were determined with reference to sample sizes and outcome variability reported in previously published studies using comparable hypertension and atrial remodeling or AF models [22,23,24,25]. Mice were randomly assigned to three groups (using an online random-number generator: https://randomtool.cn/random-group/; accessed on 9 August 2025) and received the following treatments:

Control group: subcutaneous implantation of a saline-filled osmotic mini-pump and an intraperitoneal saline injection every other day for 21 days.

Ang II group: subcutaneous implantation of an Ang II-filled osmotic mini-pump and an intraperitoneal saline injection every other day for 21 days.

Ang II + AP-III-a4 group: subcutaneous implantation of an Ang II-filled osmotic mini-pump and an intraperitoneal injection of AP-III-a4 (20 mg/kg) every other day for 21 days.

Mice were monitored daily throughout the study for general condition, activity, food and water intake, and signs of pain or distress. Those reaching the predefined humane endpoints, including >20% body-weight loss, inability to eat or drink, severe lethargy, respiratory distress, or unrelieved pain, were immediately anesthetized and euthanized as described below. At the planned experimental endpoint or upon reaching a humane endpoint, mice and rats were deeply anesthetized with sodium pentobarbital (50 mg/kg, intraperitoneally). After the absence of the pedal withdrawal reflex was confirmed, animals were euthanized by cervical dislocation performed by trained personnel. Death was confirmed by the absence of spontaneous respiration and heartbeat before tissue collection.

On day 22, echocardiography, electrocardiography, and AF induction were performed before euthanasia. Hearts were excised, and the left atria were dissected and washed with PBS. Tissue samples were either snap-frozen in liquid nitrogen for protein/RNA extraction, sequencing, Western blotting, and qPCR, or fixed in 4% paraformaldehyde for paraffin or frozen sectioning followed by hematoxylin–eosin, Masson, and immunofluorescence staining. Investigators who performed echocardiographic and electrophysiological assessments, as well as those who quantified histological and fluorescence images, were blinded to group allocation. Western blotting and RT-qPCR experiments, including their quantification, were not performed in a blinded manner. Every effort was made to minimize animal suffering and the number of animals used.

### 2.3. Electrophysiological Assessment

#### 2.3.1. In Vivo AF Induction in Rats

In vivo electrophysiological recording and AF induction in rats were performed using an Electrical Mapping System (Scope Technology, Hangzhou, China). The rats were anesthetized with pentobarbital, and surface ECGs were recorded for 5 min. The right jugular vein was exposed, and an octopolar electrophysiology catheter was inserted. The catheter position was determined using intracardiac electrograms. The pacing threshold was established using 1 ms pulses at a rate 10 beats/min above the basal heart rate; capture was confirmed by a QRS complex after each stimulus. Subsequent pacing was delivered at twice the threshold. AF was induced with continuous burst pacing (S1S1, 20 ms cycle length, 1 ms pulse width, 10 s) and repeated five times after recovery of the sinus rhythm. AF was defined as an episode lasting >1 s [22,26], characterized on surface electrocardiography by the absence of discernible P waves and an irregular R-R interval. To minimize the misclassification of transient post-pacing responses or pacing artifacts, AF was considered inducible only when an episode meeting these criteria occurred in at least three of five pacing attempts. For animals meeting the predefined AF-inducibility criterion, AF duration was calculated as the mean duration of the AF episodes induced among the successfully induced episodes. Animals that did not meet the inducibility criterion were assigned an AF duration of 0 s.

#### 2.3.2. ECG Recording and AF Induction in Mice

In vivo ECG recording and transjugular programmed electrical stimulation in mice were performed using the ADInstruments system (Sydney, Australia), following a procedure similar to that used in rats. Under anesthesia, baseline surface ECGs were recorded, followed by placement of a Millar octapolar catheter through the jugular vein into the upper right atrium. A pacing-induced AF episode was defined as an event lasting at least 1 s with loss of discernible sinus P waves and an irregularly irregular R-R interval after the final pacing stimulus [22,27]. Post-pacing tachyarrhythmias with discernible P waves and regular R-R intervals were excluded, and AF duration was measured from the end of the final pacing stimulus to the first subsequent sinus beat. AF was considered inducible only when an episode meeting these criteria occurred in at least three of five pacing attempts. The AF inducibility and duration were compared among groups. ECG data were analyzed using LabChart 8 (ADInstruments, Sydney, Australia). The atrial effective refractory period (AERP) was determined using an S1–S2 programmed-stimulation protocol. Eight consecutive S1 stimuli were delivered at a basic cycle length of 100 ms, followed by a premature S2 stimulus. The S1–S2 coupling interval was progressively shortened from 70 to 20 ms in 2 ms steps. AERP was defined as the longest S1–S2 coupling interval at which S2 failed to elicit a local atrial electrogram [28]. The duration of induced AF in mice was calculated using the same criteria as in the rat experiments.

### 2.4. Noninvasive Blood Pressure Measurement

Blood pressure was measured noninvasively using an ADInstruments NIBP system (Sydney, Australia) [29]. All animals were acclimated to the restraint sleeve for one week before testing. Animals were placed in the sleeve with their tails exposed and maintained on a 37 °C heating pad. An occlusion cuff and VPR sensor cuff were placed at the tail base. Once the tail was warmed and the animal was stable, 10 consecutive blood-pressure measurements were obtained at each assessment time point, and the mean value was used for analysis.

### 2.5. Echocardiography

Rat echocardiography was performed with a Vevo 2000 small-animal ultrasound system (FUJIFILM VisualSonics, Toronto, ON, Canada). Chest hair was removed the day before examination. Following isoflurane induction, parasternal long-axis B- and M-mode images and standard four-chamber B-mode images were acquired. The measurements included left-ventricular anterior and posterior wall thickness (LVAW and LVPW), heart rate (HR), left ventricular mass (LV mass), end-systolic and end-diastolic volumes, ejection fraction (EF), fractional shortening (FS), maximal left atrial diameter, and left atrial area.

Echocardiographic assessment in mice was performed using a Vevo 3100 small-animal ultrasound imaging system (FUJIFILM VisualSonics, Toronto, ON, Canada). The imaging procedures and measurements were the same as for rats.

### 2.6. Isolation of Primary Atrial Cardiomyocytes

Neonatal rats (postnatal 1–3 days) of undetermined sex were deeply anesthetized with sodium pentobarbital (50 mg/kg, intraperitoneally). After the absence of a response to noxious stimulation was confirmed, pups were euthanized by cervical dislocation, performed by trained personnel. Death was confirmed by the absence of spontaneous respiration and heartbeat. Hearts were excised; atrial tissue was retained, washed in pre-chilled D-HBSS, and minced. Tissue was incubated overnight (16–20 h) at 4 °C in 0.025% trypsin without EDTA. Serum was added to a final concentration of 10%, and the suspension was warmed at 37 °C for 10 min. Collagenase II was added, followed by 2 min at room temperature and filtration through a 70 µm filter. The cell suspension was centrifuged at 200× *g* for 3 min, resuspended in DMEM containing 10% FBS and 1% penicillin/streptomycin, and subjected to differential adhesion for 1 h and then 90 min. The final cell suspension was supplemented with BrdU and seeded for subsequent experiments.

### 2.7. Cell Treatment Protocol

Primary atrial cardiomyocytes were cultured in DMEM supplemented with 10% FBS and 1% penicillin/streptomycin. Cells were treated with Ang II (10 µM [30,31,32]), AP-III-a4 (10 µM [21,33]), 740Y-P (10 µM [34,35,36]) or LY294002 (10 µM [27,37,38]), either alone or in the indicated combinations. After 24 h of stimulation, the cells were harvested for subsequent experiments.

### 2.8. RNA Knockdown

siRNA oligonucleotides targeting ENO1 were designed and synthesized by GenePharma (Shanghai, China). A scrambled RNA duplex served as the negative control. The si-ENO1 sequences were as follows: sense (5′–3′), CGAGUCUCUGCAGGCGUGUTT; antisense (5′–3′), ACACGCCUGCAGAGACUCGTT.

For transfection in six-well plates, 2 µL of siRNA, 6 µL of Lipofectamine RNAiMAX, and 600 µL of Opti-MEM were added per well. The medium was replaced with complete medium after 8 h. Knockdown efficiency was evaluated by Western blotting and qPCR 36 h after transfection, after which cells were used for subsequent experiments.

### 2.9. Histopathological Examination

After perfusion with ice-cold PBS, cardiac tissues were fixed in 4% paraformaldehyde for at least 24 h, dehydrated through a graded series, embedded in paraffin, and sectioned at 5 µm. Hematoxylin–eosin, Masson trichrome, and Sirius red staining were performed to assess histopathological changes. Images were acquired using light microscopy and subjected to quantitative analysis.

### 2.10. DHE Staining for ROS Detection

Primary cardiomyocytes were treated with Ang II for 24 h, washed twice with PBS, and incubated with 25 µM DHE for 30 min at 37 °C in the dark. After three PBS washes, nuclei were stained with Hoechst (10 µg/mL) for 5 min. For atrial tissue, frozen sections were washed with PBS for 10 min, incubated with DHE for 30 min at 37 °C in the dark, washed, and mounted with DAPI-containing medium. All images were acquired by IX73 P1F fluorescence microscopy (Olympus, Tokyo, Japan).

### 2.11. Mitochondrial Membrane Potential Measurement

JC-1 is widely used to assess mitochondrial membrane potential. At high membrane potential, JC-1 accumulates in the mitochondrial matrix as J-aggregates and emits red fluorescence; at low membrane potential, it remains monomeric and emits green fluorescence. After stimulation, cells were washed three times with PBS and incubated with JC-1 (10 µg/mL) for 20 min at 37 °C in the dark. The nuclei were stained with Hoechst, and the red-to-green fluorescence intensity ratio was quantified by fluorescence microscopy.

### 2.12. Measurement of Mitochondrial ROS Production

Mitochondrial ROS production in primary atrial cardiomyocytes was assessed with MitoSOX Red and MitoTracker Green according to the manufacturers’ instructions. Working solutions were prepared in serum-free medium at final concentrations of 5 µM MitoSOX Red and 200 nM MitoTracker Green. After stimulation, cells were washed with PBS, incubated with dye for 20 min at 37 °C in the dark, washed three times, stained with Hoechst for 5 min, washed again, and imaged by fluorescence microscopy.

### 2.13. Immunofluorescence Staining

After fixation in paraformaldehyde for 24 h, tissues were dehydrated in 30% sucrose for 24 h, embedded in OCT, and cryosectioned at 7 µm. Sections were equilibrated for 20 min at room temperature, washed to remove OCT, and blocked/permeabilized for 15 min with QuickBlock™ Immunostaining Blocking Solution (Beyotime Biotechnology, Shanghai, China). Primary antibodies diluted in SignalUp™ Immunostaining Signal Enhancer (Beyotime Biotechnology, Shanghai, China) were incubated overnight at 4 °C. Sections were washed three times with PBS, incubated with secondary antibodies for 60 min at room temperature, mounted with DAPI-containing antifade medium, and examined by Nikon A1 confocal laser-scanning microscope (Nikon, Tokyo, Japan).

### 2.14. Protein Extraction and Western Blotting

RIPA buffer was mixed with protease and phosphatase inhibitors at 100:1 to prepare lysis buffer. Atrial tissues or cells were lysed in lysis buffer. After centrifugation, the supernatants were collected, and protein concentrations were quantified using a bicinchoninic acid (BCA) protein assay kit. Proteins were separated by 4–20% SDS-PAGE and transferred to PVDF membranes. After blocking with 5% BSA for 120 min at room temperature, membranes were incubated with primary antibodies overnight at 4 °C. The membranes were then washed with TBST and incubated with HRP-conjugated secondary antibodies (1:5000) for 90 min. Chemiluminescence was detected using an ECL kit. Protein expression was quantified by ImageJ (version 1.54d; National Institutes of Health, Bethesda, MD, USA) densitometry, with β-actin as the internal control.

### 2.15. RNA Extraction and RNA Sequencing

RNA sequencing was performed using left atrial tissues collected from 12- and 24-week-old SHRs and age-matched WKY rats (*n* = 4 per group). Total RNA was extracted from left atrial tissues using TRIzol reagent. Then RNA quality was determined using Agilent 5300 Fragment Analyzer (Agilent Technologies, Santa Clara, CA, USA) and quantified using the ND-2000 (NanoDrop Technologies, Wilmington, DE, USA). Only RNA samples meeting the specified quality criteria (concentration ≥ 20 ng/µL, total RNA > 1 µg, RQN > 4.5) were used for sequencing-library preparation.

Sequencing libraries were prepared using the Illumina^®^ Stranded mRNA Prep (Illumina, San Diego, CA, USA) and sequenced by Majorbio Bio-Pharm Technology Co., Ltd. (Shanghai, China) on the NovaSeq X Plus to generate 2 × 150 bp paired-end reads. Adapter trimming and quality filtering were performed using fastp v0.23.4 with default parameters. The 12-week dataset yielded a mean of 43.98 million clean reads per sample (range, 41.23–46.57 million; 6.13–6.87 Gb), whereas the 24-week dataset yielded a mean of 44.25 million clean reads per sample (range, 41.67–47.45 million; 6.25–7.11 Gb).

Clean reads were aligned in strand-specific mode to the *Rattus norvegicus* mRatBN7.2 reference genome (NCBI RefSeq assembly accession GCF_015227675.2; annotation release GCF_015227675.2-RS_2023_06) using HISAT2 v2.2.1. Reference-guided transcript reconstruction was performed using StringTie v2.2.1. RSEM v1.3.3 was used separately to estimate gene-level expression and generate count estimates and transcripts-per-million (TPM) values. The RSEM-derived gene-level expected-count matrix was supplied to DESeq2 v1.42.0 using the DESeqDataSetFromMatrix function. TPM values were used only for expression visualization and were not used for differential-expression testing. The StringTie transcript assemblies were not used as the expression matrix for differential-expression testing. The 12- and 24-week cohorts were analyzed separately in DESeq2 v1.42.0 using the design formula ~ group (SHR versus WKY), with group as the sole design variable; no additional biological or technical covariates were included.

Downstream visualization and enrichment analyses of the 12- and 24-week datasets were performed using the Majorbio Cloud Platform (https://cloud.majorbio.com/; accessed on 10 July 2025). Differential expression analysis was performed using DESeq2. *p* values were adjusted for multiple testing using the Benjamini–Hochberg method. Genes with an adjusted *p* < 0.05 and an absolute log2 fold change ≥ 0.58 were considered DEGs. In addition, functional enrichment analysis with GO (Version 2023.07) and KEGG (Version 2023.09) was performed to identify which DEGs were significantly enriched in GO terms and metabolic pathways at Bonferroni-adjusted *p* value < 0.05 compared with the whole-transcriptome background. GO functional enrichment and KEGG pathway analysis were carried out by GOATOOLS (Version 1.4.4) and Python scipy software (version 1.14.1), respectively.

The raw sequencing reads, processed gene-level count matrices, normalized expression matrices, and sample metadata have been deposited in the Gene Expression Omnibus (GEO) database under accession numbers GSE346715 (12-week) and GSE348072 (24-week).

### 2.16. Real-Time Quantitative PCR (RT-qPCR)

The RNA concentration was measured with a NanoDrop 2000. cDNA was synthesized using a reverse-transcription kit (AG11706, Accurate Biology, Changsha, China). RT-qPCR was performed on a QuantStudio 6 Flex instrument (Thermo Fisher Scientific, Waltham, MA, USA) using qPCR SYBR Green Master Mix (11201ES03, Yeasen, Shanghai, China). Relative gene expression was calculated using the 2^−ΔΔCt^ method. Primers were supplied by Tsingke Biotech (Beijing, China) and are listed in Supplementary Table S1.

### 2.17. Quantitative Proteomic Analysis

Primary atrial cardiomyocytes were transfected with negative-control siRNA (si-NC) or ENO1-targeting siRNA (si-ENO1) and subsequently stimulated with Ang II for 24 h. The si-NC + Ang II group served as the control group. Four biologically independent samples per group were subjected to quantitative proteomic analysis. Cells were lysed in buffer containing 8 M urea and 1% protease and phosphatase inhibitor mixture, and protein concentration was determined using a BCA assay. Equal amounts of protein from each biological replicate were reduced with DTT, alkylated with IAA, digested with trypsin, and desalted using Strata X cartridges (Phenomenex, Torrance, CA, USA).

Peptides were separated using a Vanquish Neo nano-flow UHPLC system and analyzed on an Orbitrap Astral mass spectrometer (Thermo Fisher Scientific, Waltham, MA, USA) in positive-ion data-independent acquisition (DIA) mode. MS1 spectra were acquired over an *m*/*z* range of 380–980 at a resolution of 240,000 at *m*/*z* 200, with a normalized automatic gain control target of 500% and a maximum injection time of 5 ms. MS2 spectra were acquired in DIA mode using 299 windows with an isolation width of 2 *m*/*z*, a higher-energy collisional dissociation energy of 25 eV, a normalized automatic gain control target of 500%, and a maximum injection time of 3 ms.

Raw DIA data were processed using DIA-NN (version 1.8.1) against the UniProtKB Rattus norvegicus reference proteome database (release 2024_06). Database searching used trypsin specificity, allowing up to one missed cleavage, with carbamidomethylation of cysteine as a fixed modification and methionine oxidation and protein N-terminal acetylation as variable modifications. Protein identifications from database searching were filtered at an FDR < 1%.

Protein intensities were log2-transformed, protein-wise mean-centered, and subsequently median-normalized on a sample-wise basis. Missing values were imputed using the k-nearest-neighbor algorithm implemented in the Bioconductor impute package (impute::impute.knn 1.78.0; k = 10; rowmax = 0.5, colmax = 0.8, maxp = 1500, rng.seed = 362,436,069). Within the vendor workflow, only low-abundance values classified as missing not at random were imputed; values considered missing at random were not imputed. Differential protein abundance was assessed using limma (3.64.0) with empirical-Bayes moderated t statistics. *p* values were adjusted across all tested proteins using the Benjamini–Hochberg method to control the FDR. Differentially expressed proteins (DEPs) were defined as proteins with an adjusted *p* value < 0.05 and an absolute log2 fold change ≥ 0.58. For GO and KEGG enrichment analyses, Fisher’s exact-test *p* values were adjusted using the Benjamini–Hochberg procedure across the tested terms or pathways, and an adjusted *p* value < 0.05 was considered significant. Proteomic experiments and subsequent bioinformatic analyses were performed by Hangzhou Cosmos Wisdom Biotech Co., Ltd. (Hangzhou, China). The mass spectrometry proteomics data have been deposited to the ProteomeXchange Consortium via the PRIDE partner repository with the dataset identifier PXD085550.

### 2.18. Reanalysis of Public Human Atrial-Fibrillation Datasets

GSE41177 and GSE115574 were used solely as external observational datasets to assess the association between ENO1 expression and established human AF. Both datasets were generated using the Affymetrix Human Genome U133 Plus 2.0 Array (GPL570).

For GSE41177, analysis was restricted to left-atrial tissue samples from 16 patients with persistent AF and 3 sinus-rhythm participants undergoing valvular surgery. This restriction avoided treating paired samples from the left atrial appendage and left atrial–pulmonary vein junction from the same participant as independent observations. For GSE115574, analysis was restricted to left-atrial samples from 14 patients with AF and 15 sinus-rhythm participants with chronic severe degenerative mitral regurgitation, after the exclusions reported in the source study. Thus, each retained array represented a different participant.

The processed series-matrix files deposited in GEO were used for reanalysis. The expression values had been preprocessed by the original studies using robust multi-array average normalization, including background correction, quantile normalization, and log2 transformation; no additional normalization was performed in the present study. Affymetrix probe-set identifiers were mapped to official gene symbols according to the GPL570 platform annotation file obtained from GEO. Expression differences between AF and sinus-rhythm samples were assessed using limma linear models, and *p* values were adjusted using the Benjamini–Hochberg method. Individual-level hypertension status linked to the GEO sample identifiers was unavailable for both datasets and therefore could not be included as a sample-level covariate.

### 2.19. Hydrogen Peroxide Measurement

Intracellular H_2_O_2_ was measured using a commercial detection kit (Beyotime, Shanghai, China) according to the manufacturer’s instructions. Briefly, cells were collected and lysed, 50 µL of sample was mixed with 100 µL of H_2_O_2_ detection reagent, and the mixture was incubated for 30 min at room temperature. Absorbance was measured at 560 nm, and H_2_O_2_ concentrations were calculated from the standard curve.

### 2.20. Statistical Analysis

Statistical analyses and graph generation were performed using GraphPad Prism 8 and SPSS 25. Normally distributed continuous data are presented as mean ± standard deviation (SD); non-normally distributed data are presented as median and interquartile range. All tests were two-sided, and *p* < 0.05 was considered statistically significant. Normality was assessed using the Shapiro–Wilk test, and homogeneity of variance was assessed using Levene’s test before parametric analysis. Technical replicates were averaged before statistical testing so that each data point represented one independent animal or one independent primary-cardiomyocyte preparation. For normally distributed continuous variables with homogeneous variances, comparisons between two groups were performed using an unpaired Student’s *t*-test, and comparisons among three or more groups were performed using one-way ANOVA followed by Tukey’s multiple-comparison test. For non-normally distributed continuous outcomes, including AF duration, comparisons among three or more groups were performed using the Kruskal–Wallis test followed by Dunn’s multiple-comparison test. Longitudinal blood-pressure data were analyzed using a linear mixed-effects model with group, time, and their interaction as fixed effects and animal as a random effect. Pairwise comparisons were performed using estimated marginal means with Šidák correction. A two-sided *p* < 0.05 was considered statistically significant. AF inducibility was analyzed as a binary outcome using two-sided Fisher’s exact tests for prespecified pairwise comparisons, with Holm adjustment for multiple testing. For each animal meeting the AF-inducibility criterion, AF duration was calculated as the mean duration of successfully induced AF episodes; animals not meeting the criterion were assigned 0 s and retained in the analysis. No samples were excluded; experiments were repeated independently.

## 3. Results

### 3.1. Long-Term Hypertension Promotes Atrial Remodeling and AF Inducibility in Rats

#### 3.1.1. Hypertension Promotes Cardiac Remodeling in Rats

Hypertension is a major risk factor for AF. The available experimental models include the SHR, Ang II mini-pump, L-NAME, and DOCA models. We chose SHRs for their close resemblance to human primary hypertension and for their ability to reproduce long-term disease progression. Blood pressure was greatly elevated in SHRs, with systolic blood pressure reaching approximately 180 mmHg at 12 weeks and up to 240 mmHg at 24 weeks. Diastolic blood pressure rose from 120 mmHg at 12 weeks up to 150 mmHg at 24 weeks (Figure S1A,B). At 24 weeks, the body weight, total heart weight, and heart weight/body weight ratio did not differ substantially between the SHRs and WKY rats. However, the atrial weight and atrial-to-heart-weight ratio were significantly greater in hypertensive rats than in controls (Figure S1C).

Echocardiography failed to show any major differences with regard to ejection fraction (EF), fractional shortening (FS), ventricular wall thickness, or left ventricular mass between the SHRs and WKY rats at 12 weeks (Figure S1D,E). With advancing hypertension, blood pressure increased further; the 24-week SHRs showed higher EF and FS than controls, indicating a hyperdynamic state and ventricular hypertrophy (Figure S1F,G). By contrast, the left atrial maximal diameter and area had already tended to increase at 12 weeks in the SHRs, although the differences were not statistically significant (*p* = 0.0513 and *p* = 0.0508, respectively). At 24 weeks, both the left atrial maximal diameter and area were significantly increased (Figure S2A–C). Masson’s trichrome staining showed a trend toward increased left atrial collagen deposition at 12 weeks and marked fibrosis at 24 weeks (Figure S2D,E).

These findings show that atrial enlargement was already evident as a trend during early hypertension, whereas no significant ventricular changes were observed. With the progression of hypertension, atrial remodeling became more pronounced and ventricular changes tended to follow shortly after. Such observations support the idea that structural remodeling may occur earlier in the atria than in the ventricles during the course of hypertension.

#### 3.1.2. Hypertension Increases AF Inducibility in Rats

Hypertension is known to cause definite atrial structural remodeling; we next investigated the effects of hypertension on AF inducibility and AF duration. Because spontaneous AF was not observed in rats with hypertension, we used in vivo programmed stimulation to evaluate AF inducibility. Successful AF induction was defined as an absolutely irregular rhythm without P waves lasting >1 s. At the early stage of hypertension, AF inducibility and duration tended to be slightly higher (only one extra rat in the SHR group versus the WKY group). With long-term high blood pressure, both AF inducibility and AF duration were significantly greater in SHRs than in WKY rats (Figure S2F–H).

These findings demonstrate an association between long-term hypertension, progressive atrial remodeling, and increased susceptibility to pacing-induced AF in rats.

### 3.2. ENO1 Is Markedly Upregulated in the Left Atrium of SHRs

**Figure 1:**
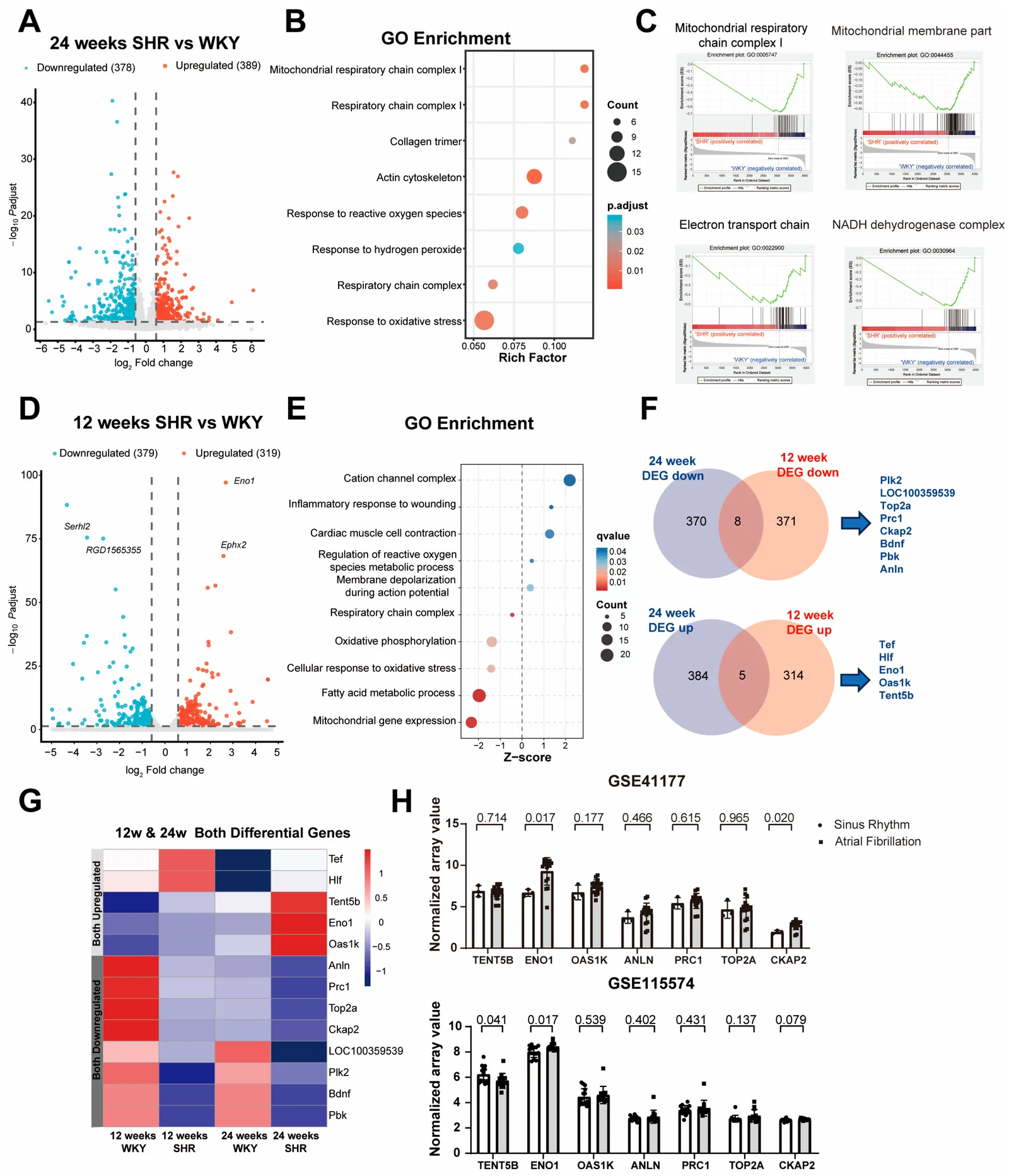
ENO1 is consistently upregulated in hypertensive rat atria and human AF datasets. (**A**) Volcano plots of mRNAs significantly upregulated or downregulated in left atrial tissues from 24-week-old SHRs compared with WKY rats. A total of 389 genes were upregulated and 378 genes were downregulated (adjusted *p* < 0.05, |log2 FC| ≥ 0.58, *n* = 4 per group). In panels A and D, red, blue, and gray points denote significantly upregulated, significantly downregulated, and nonsignificant genes, respectively; the vertical and horizontal dashed lines indicate the |log2 fold-change| and adjusted-*p*-value cutoffs. (**B**) Bubble plot showing Gene Ontology (GO) enrichment analysis of the identified DEGs. (**C**) Gene set enrichment analysis (GSEA) of the DEGs. (**D**) Volcano plot of DEGs in 12-week-old SHRs versus WKY rats. A total of 319 genes were upregulated and 379 genes were downregulated (adjusted *p* < 0.05, |log2 FC| ≥ 0.58, *n* = 4 per group). (**E**) Z-score bubble plot of GO enrichment analysis of the DEGs, dashed lines indicate Z-score = 0; values were normalized in the upward direction. (**F**) Venn diagram showing genes that were consistently upregulated or downregulated in both the 12- and 24-week rats. (**G**) Heatmap showing the expression profiles of genes with concordant changes at 12 and 24 weeks. (**H**) Expression of candidate key genes in human left atrial tissue datasets from patients with AF (GSE41177 and GSE115574). Data are shown as mean ± SD.

Because AF inducibility was significantly increased in 24-week-old SHRs, we examined their atrial transcriptomic profiles. To investigate the potential role of hypertension in promoting AF, we performed transcriptomic analysis of left atrial tissues from 24-week-old SHRs and WKY rats. The volcano plot analysis identified 767 DEGs, comprising 389 upregulated and 378 downregulated genes (adjusted *p* < 0.05; |log2FC| ≥ 0.58) (Figure 1A). Multiple pathways related to mitochondrial energy production and ROS/H_2_O_2_ generation, including pathways involving mitochondrial respiratory-chain complexes and cellular responses to ROS or hydrogen peroxide, were significantly altered in SHRs (Figure 1B). The GSEA revealed marked suppression of pathways related to mitochondrial respiratory complex I, the electron transport chain, the NADH dehydrogenase complex, and mitochondrial membrane organization in hypertensive left atria, which points toward mitochondrial dysfunction and increased ROS generation under hypertension (Figure 1C).

Hypertension is a chronic disease, and its consequences for tissues seem unlikely to be temporary. Identifying and targeting early pathogenic mechanisms of hypertension is important for reducing the incidence of hypertension-associated AF. Thus, we performed transcriptomic analysis of left atrial tissues from 12-week SHRs and WKY rats. In early hypertension (12 weeks), 319 genes were upregulated and 379 were downregulated (Figure 1D). The Z-score and GO methods showed an overall reduction in oxidative phosphorylation, respiratory complex pathways, and several important mitochondrial metabolic processes which were associated with an increase in ROS and cardiac electrical activity in the early stages of hypertension (Figure 1E). Because hypertension is a chronic and progressive condition, we hypothesized that its effects on the atria would be sustained over time. Accordingly, candidate targets were expected to be altered at both 12 and 24 weeks, with consistent directions of change at the two time points (i.e., either upregulated or downregulated at both time points). We intersected genes altered at 12 and 24 weeks and identified eight consistently downregulated and five consistently upregulated genes (Figure 1F). The heatmap illustrates the expression profiles of these genes across the different groups (Figure 1G). Because hypertension leads to long-term injury, it seems reasonable to expect that its effect on the left atrium would become progressively worse. On this basis, only seven genes exhibited expression patterns consistent with progressive changes: TENT5B, ENO1, OAS1K, ANLN, PRC1, TOP2A, and CKAP2. Hypertension is one of the most important risk factors for AF. To further investigate whether candidate genes related to hypertension are associated with human AF, we analyzed two publicly available human left atrial tissue transcriptomic datasets of AF (GSE41177 and GSE115574) to evaluate the expression of candidate genes identified in the hypertensive rat model. Among the candidate genes examined, ENO1 was the only gene significantly upregulated in AF samples in both datasets (Figure 1H), with a direction of change consistent with that observed in the atria of hypertensive rats. This cross-species, cross-disease consistency has identified ENO1 as a candidate gene associated with hypertensive atrial remodeling and human AF.

**Figure 2:**
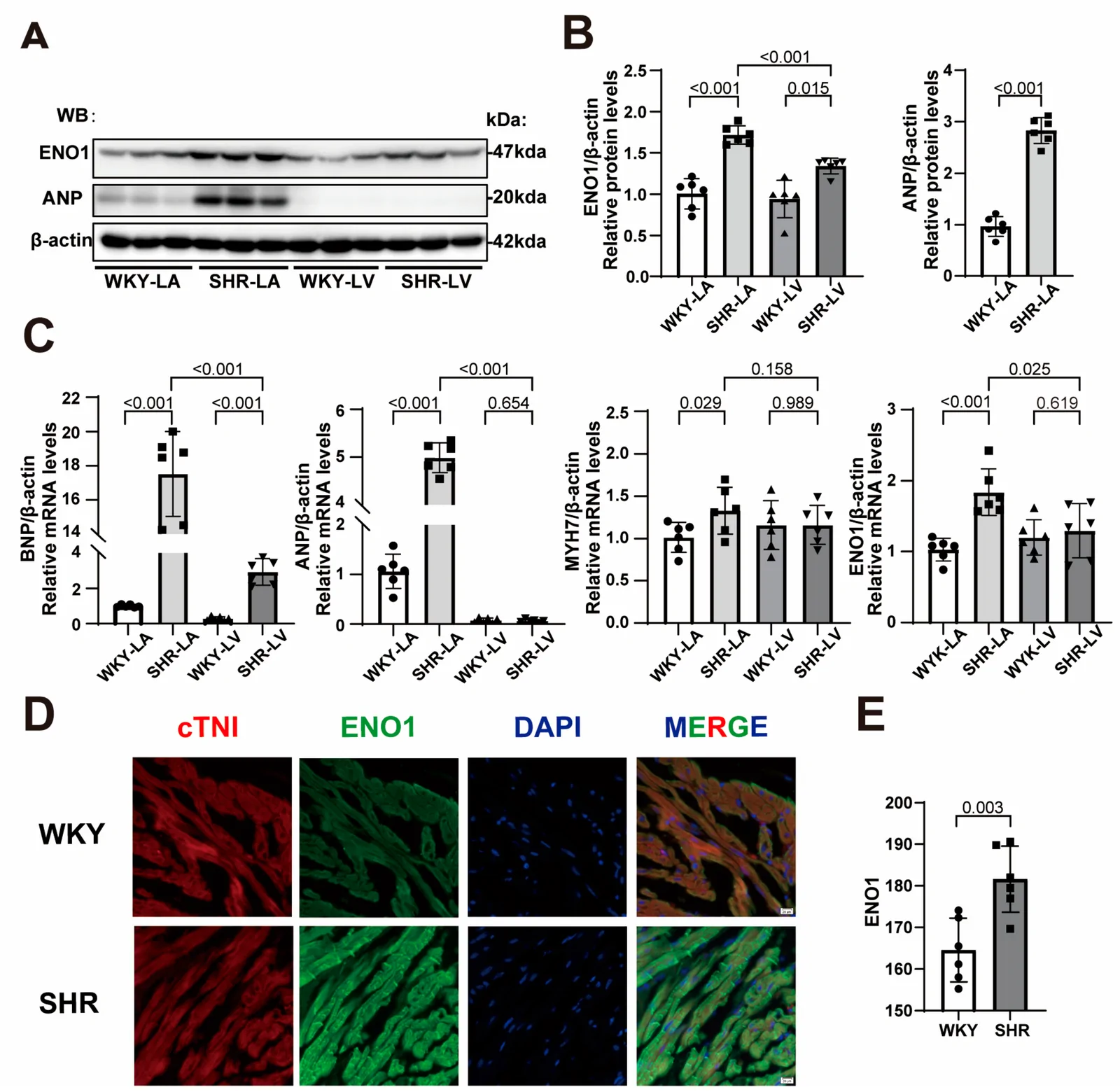
ENO1 is highly expressed in the atrial myocardium of hypertensive rats. (**A**,**B**) Western blot analysis and quantification of ENO1 and ANP expression in atrial and ventricular tissues from 12-week-old SHRs and WKY rats (*n* = 6 per group). (**C**) RT-qPCR analysis of BNP, ANP, MYH7, and ENO1 expression in atrial and ventricular tissues from 12-week-old hypertensive rats (*n* = 6 per group). (**D**,**E**) Representative immunofluorescence colocalization analysis showing the expression and localization of ENO1 in rat atrial tissue. The corresponding quantification shows mean fluorescence intensity (*n* = 6 per group). Data are shown as mean ± SD. Scale bar: 20 µm.

The transcriptomic findings were subsequently evaluated at the protein and mRNA levels. ENO1 expression was significantly higher in the atria of 12-week-old SHRs than in those of WKY rats and was greater in atrial than in ventricular tissue (Figure 2A–C). Immunofluorescence colocalization indicated that ENO1 was mainly present in atrial cardiomyocytes and was significantly increased in SHR atria compared to WKY atria (Figure 2D,E and Figure S3A–C).

The transcriptomic analyses identified changes in mitochondria- and redox-related pathways at both early and later stages of hypertension. Longitudinal intersection analysis identified several genes, including ENO1, that exhibited persistent changes across disease stages. Among the candidate genes evaluated, ENO1 was the only gene significantly upregulated in both external human AF datasets. Collectively, these findings nominate ENO1 as a candidate associated with hypertension-related atrial remodeling and with established human AF.

### 3.3. siRNA-Mediated ENO1 Knockdown and AP-III-a4 Treatment Attenuate Ang II-Induced Atrial Injury

**Figure 3:**
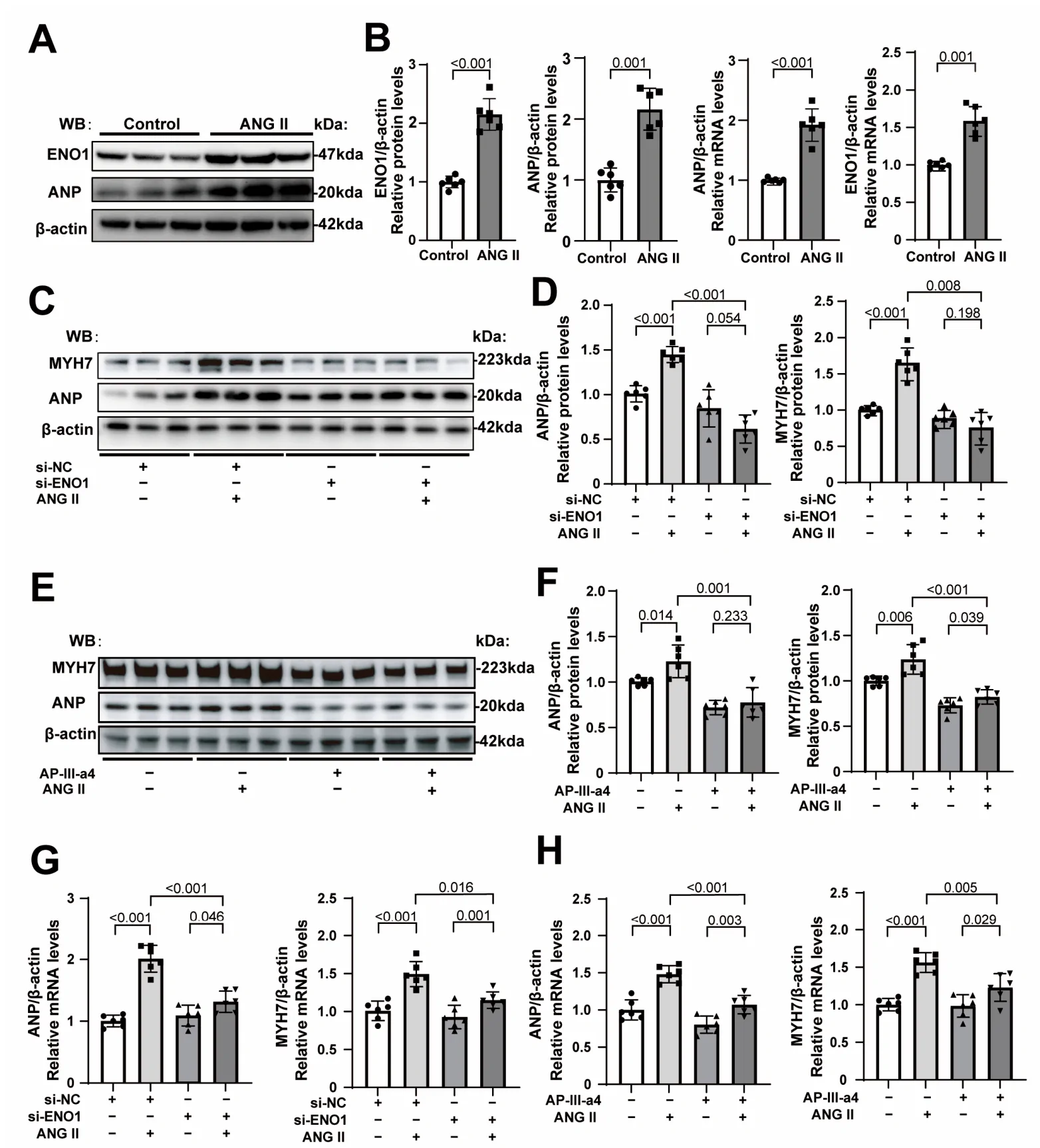
ENO1 knockdown and AP-III-a4 treatment attenuate Ang II-induced atrial cardiomyocyte injury. (**A**,**B**) Western blot and RT-qPCR analyses of ENO1 expression in atrial cardiomyocytes following Ang II stimulation (*n* = 6 per group). (**C**,**D**) Effects of siRNA-mediated ENO1 knockdown on Ang II-induced ANP and MYH7 expression at the protein level in atrial cardiomyocytes (*n* = 6 per group). (**E**,**F**) Effects of AP-III-a4 treatment on Ang II-induced ANP and MYH7 protein expression in atrial cardiomyocytes (*n* = 6 per group). (**G**,**H**) Effects of ENO1 knockdown or AP-III-a4 treatment on Ang II-induced MYH7 and ANP mRNA expression in primary atrial myocytes (*n* = 6 per group). Data are presented as mean ± SD.

ENO1 was highly expressed in the left atrial tissue of SHRs. To investigate its function in hypertensive atrial cardiomyocytes, we isolated primary neonatal rat atrial cardiomyocytes. Establishing a matched cellular model directly derived from SHRs is technically challenging. Ang II is the principal effector peptide of the renin–angiotensin–aldosterone system and is widely used to induce cardiomyocyte hypertrophic and oxidative-stress responses relevant to hypertensive atrial remodeling. Accordingly, primary neonatal rat atrial cardiomyocytes were exposed to Ang II to establish an in vitro model of atrial cardiomyocyte stress and injury relevant to hypertensive atrial remodeling [22,39]. Western blotting and qPCR showed marked upregulation of the hypertrophic marker ANP and ENO1 after Ang II stimulation at both the protein and mRNA levels (Figure 3A,B), consistent with the hypertensive rat models.

To examine the role of ENO1 in Ang II-stimulated atrial cardiomyocytes, ENO1 was silenced using siRNA. Following the validation of knockdown efficiency (Figure S4A–C), siRNA-ENO1 (si-ENO1) was used in primary atrial cardiomyocytes. Following Ang II stimulation, the ANP and MYH7 expression levels were significantly lower at the protein level in the ENO1-knockdown group than in the Ang II control group (Figure 3C,D). AP-III-a4 was initially reported as an enolase-targeting compound [40]. Consistent with the direction observed after siRNA-mediated ENO1 knockdown, cells co-treated with AP-III-a4 and Ang II exhibited significantly lower ANP and MYH7 expression than cells treated with Ang II alone (Figure 3E,F). This effect was observed not only at the protein level but also at the transcriptional level, in which both AP-III-a4 treatment and siRNA-mediated ENO1 knockdown significantly attenuated Ang II-induced cardiomyocyte injury (Figure 3G,H).

**Figure 4:**
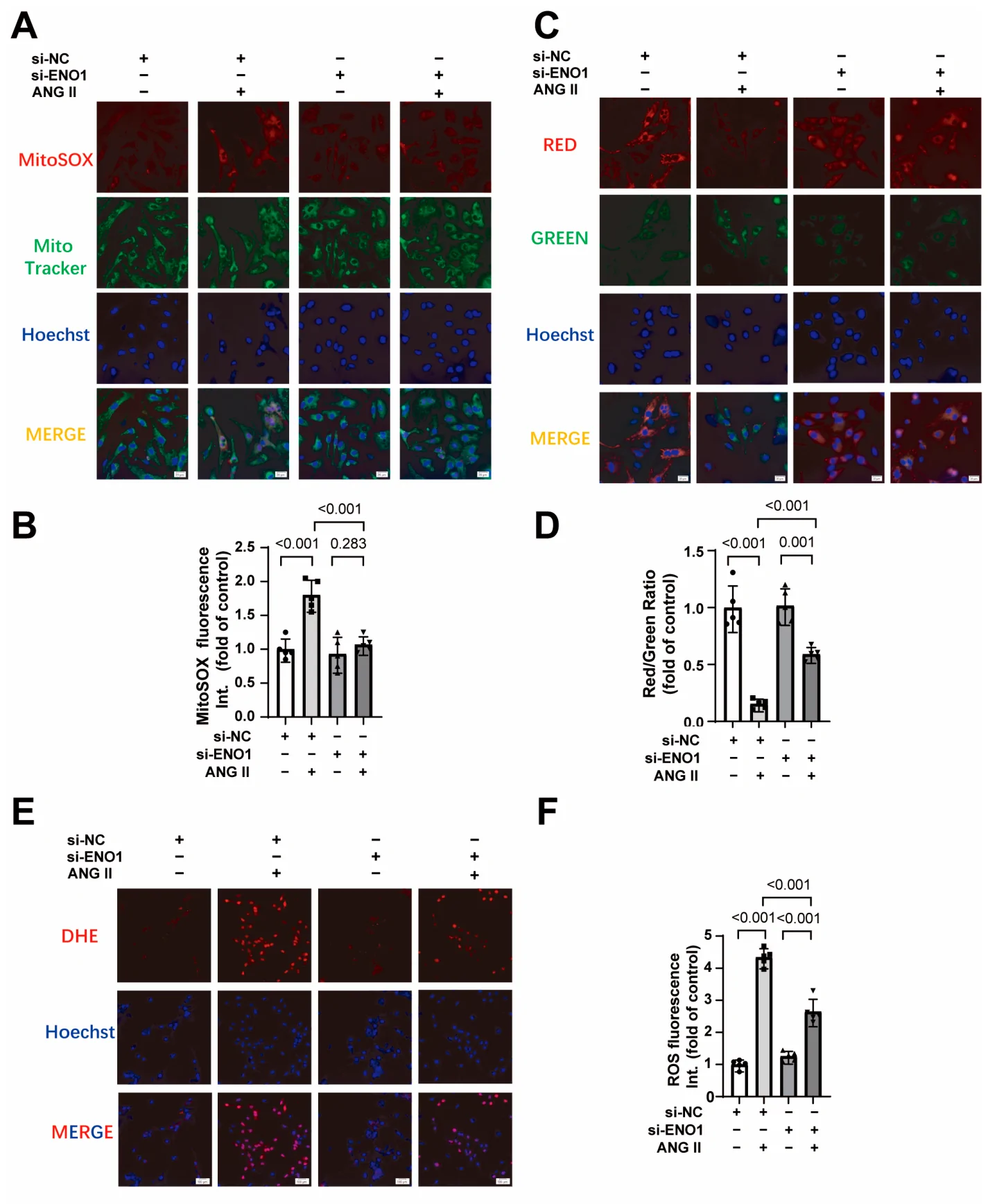
ENO1 knockdown attenuates Ang II-induced oxidative stress in atrial cardiomyocytes. (**A**,**B**) Representative MitoSOX and MitoTracker staining images and corresponding quantitative analyses (*n* = 5 per group). Scale bar: 20 μm. (**C**,**D**) Representative JC-1 staining images and corresponding quantitative analyses (*n* = 5 per group). Scale bar: 20 μm. (**E**,**F**) Representative dihydroethidium (DHE) fluorescence staining images and corresponding quantitative analyses (*n* = 5 per group). Data are presented as mean ± SD. Scale bar: 100 μm.

Hypertensive transcriptomic data indicated extensive mitochondrial injury, mitochondrial membrane disruption, and ROS accumulation in hypertensive atria. MitoSOX and MitoTracker colocalization showed that ENO1 knockdown reduced Ang II-induced mitochondrial superoxide accumulation (Figure 4A,B). JC-1 staining showed that Ang II decreased the red-to-green fluorescence ratio, indicating mitochondrial membrane depolarization. ENO1 knockdown partially restored this ratio, suggesting the attenuation of Ang II-induced mitochondrial dysfunction (Figure 4C,D). DHE staining further showed lower ROS-associated fluorescence in the ENO1-knockdown group than in the Ang II group (Figure 4E,F).

Together, these results show that ENO1 silencing attenuated Ang II-induced ROS accumulation, mitochondrial superoxide production, membrane depolarization, and hypertrophic responses in primary atrial cardiomyocytes. AP-III-a4 treatment produced concordant effects on the hypertrophic markers examined.

### 3.4. PI3K/AKT Signaling Participates in ENO1-Associated Changes in NOX4 Expression

**Figure 5:**
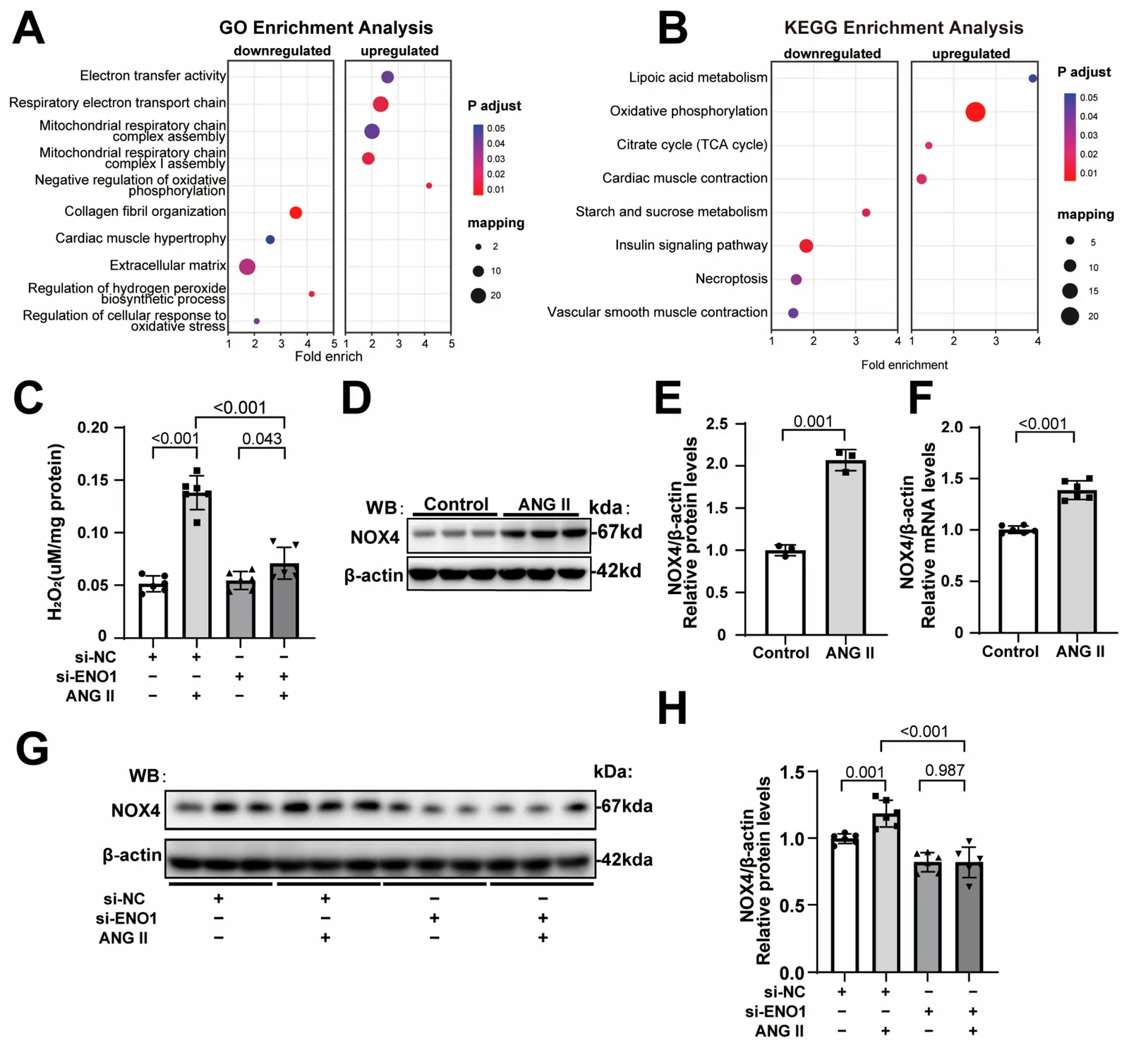
ENO1 knockdown is associated with lower NOX4 expression and intracellular H_2_O_2_ levels. (**A**) Primary atrial cardiomyocytes transfected with si-ENO1 or control siRNA were stimulated with Ang II for 24 h and subjected to quantitative proteomic analysis. The bubble plot shows GO enrichment results for DEPs in ENO1-knockdown versus control cells. (**B**) Bubble plot showing KEGG pathway enrichment results for DEPs between Ang II-stimulated ENO1-knockdown and control cells. (*n* = 4 biologically independent samples per group) (**C**) Intracellular hydrogen peroxide levels in the different cell groups (*n* = 6 per group). (**D**,**E**) Western blot analysis and quantification of NOX4 protein expression in atrial cardiomyocytes following Ang II stimulation (*n* = 3 per group). (**F**) RT-qPCR analysis of NOX4 mRNA expression in atrial cardiomyocytes following Ang II stimulation (*n* = 6 per group). (**G**,**H**) Western blot analysis and quantification of NOX4 protein expression after siRNA-mediated ENO1 knockdown in Ang II-stimulated atrial cardiomyocytes (*n* = 6 per group). Data are presented as mean ± SD.

To explore how ENO1 regulates intracellular ROS production, proteomic analysis was performed in Ang II-treated ENO1-knockdown and control atrial cardiomyocytes. GO enrichment analysis identified changes in pathways related to mitochondrial respiratory-chain complex I, electron transport, hydrogen peroxide metabolism, and ROS biosynthesis after ENO1 knockdown (Figure 5A). These pathway-level findings were consistent with alterations in mitochondrial and redox-related processes. The KEGG enrichment analysis also identified oxidative phosphorylation, lipid metabolism, the tricarboxylic acid cycle, and cardiac-muscle contraction among the altered pathways (Figure 5B). Proteomic data therefore suggested that ENO1 knockdown improves oxidative phosphorylation and reduces ROS and hydrogen peroxide accumulation. As expected, ENO1 knockdown lowered intracellular hydrogen peroxide levels (Figure 5C). NOX enzymes are important intracellular sources of reactive oxygen species. NOX enzymes transfer electrons from NADPH to molecular oxygen to generate superoxide, which can subsequently be converted into hydrogen peroxide and other ROS. Higher NOX expression, especially NOX4, has been implicated as an important source of atrial ROS during AF [41]. In patients with AF, elevated atrial NOX4 has been associated with ROS accumulation, cardiomyocyte injury, electrical and structural remodeling, and AF [42]. Oxidative stress also contributes to hypertensive target organ injury [43]; RAAS activation and related sodium/water retention can increase NOX4 expression and tissue ROS accumulation [44]. Ang II stimulation increased NOX4 expression in atrial cardiomyocytes at both the protein and mRNA levels (Figure 5D–F), whereas ENO1 knockdown reduced Ang II-induced NOX4 expression (Figure 5G,H).

**Figure 6:**
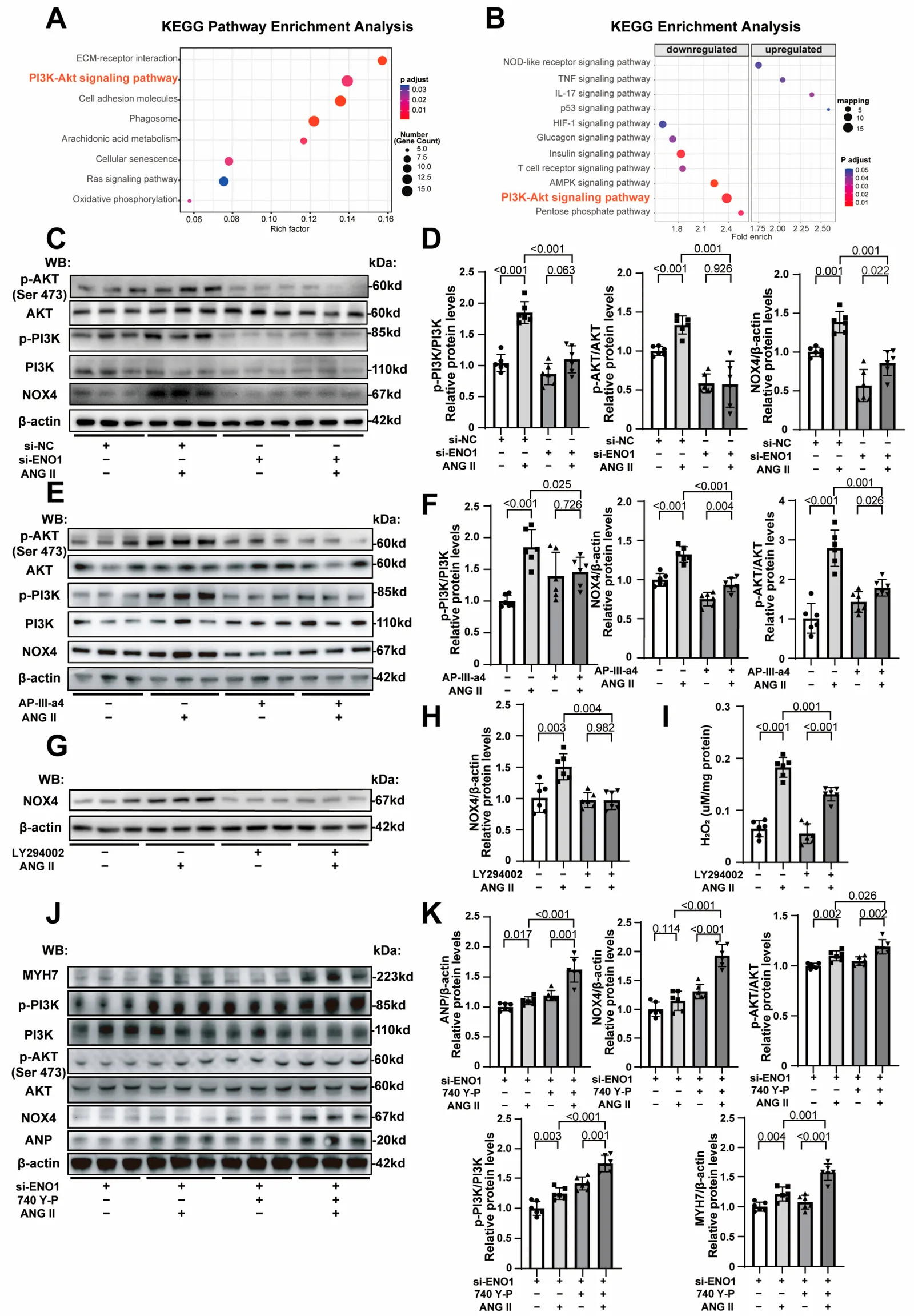
PI3K/AKT signaling participates in ENO1-associated changes in NOX4 expression. (**A**) KEGG pathway enrichment analysis of DEGs identified by atrial transcriptomic analysis in 12-week-old hypertensive and control rats. (**B**) Bubble plot showing KEGG pathway enrichment analysis of DEPs in si-ENO1 and control atrial cardiomyocytes following Ang II stimulation (*n* = 4 biologically independent samples per group). (**C**,**D**) Western blot analysis and quantification of total AKT, phosphorylated AKT (p-AKT), total PI3K, phosphorylated PI3K (p-PI3K), and NOX4 in Ang II-stimulated atrial cardiomyocytes after ENO1 knockdown (*n* = 6 per group). (**E**,**F**) Western blot analysis and quantification of total AKT, p-AKT, total PI3K, p-PI3K, and NOX4 in Ang II-stimulated atrial cardiomyocytes treated with AP-III-a4 (*n* = 6 per group). (**G**,**H**) Western blot analysis and quantification of NOX4 protein expression in atrial cardiomyocytes treated with Ang II and the PI3K inhibitor LY294002 (*n* = 6 per group). (**I**) Intracellular hydrogen peroxide levels in atrial cardiomyocytes treated with Ang II and the PI3K inhibitor LY294002 (*n* = 6 per group). (**J**,**K**) Western blot analysis and quantification of total AKT, p-AKT, total PI3K, p-PI3K, NOX4, ANP and MYH7 in primary atrial cardiomyocytes transfected with si-ENO1, stimulated with Ang II, and treated with the PI3K activator 740Y-P (*n* = 6 per group). Data are presented as mean ± SD.

In order to more comprehensively determine how ENO1 influences NOX4, KEGG enrichment was performed using DEGs from 12-week hypertensive rat atria and omics data from ENO1-knockdown primary cardiomyocytes, respectively (Figure 6A,B). The PI3K/AKT pathway was strongly enriched in 12-week hypertensive rats and greatly attenuated after ENO1 knockdown. Previous studies indicate that NOX4 expression can be regulated by PI3K/AKT signaling [45] and that increased PI3K/AKT phosphorylation can increase cellular NOX4 expression. ENO1 has already been reported as a factor activating PI3K/AKT signaling in cases of drug-induced pulmonary hypertension, with vascular smooth muscle damage as one consequence [33]. We therefore hypothesized that, in Ang II-induced atrial cardiomyocytes, ENO1 could activate PI3K/AKT signaling and thereby affect NOX4 expression.

To test this hypothesis, PI3K, p-PI3K, AKT, p-AKT, and NOX4 were measured after Ang II stimulation of ENO1-knockdown cardiomyocytes. We observed that siRNA-mediated ENO1 knockdown significantly inhibited the activation of the PI3K/AKT pathway and reduced NOX4 expression in primary atrial cardiomyocytes (Figure 6C,D). We next assessed AP-III-a4 treatment in Ang II-stimulated atrial cardiomyocytes and noted that it could markedly lower the phosphorylation of PI3K/AKT pathway components caused by Ang II, as well as the level of NOX4 (Figure 6E,F). To further elucidate the role of the PI3K/AKT pathway in regulating NOX4, we treated atrial cardiomyocytes with a PI3K inhibitor (LY294002). PI3K inhibition significantly suppressed NOX4 expression (Figure 6G,H) and also reduced the intracellular hydrogen peroxide content (Figure 6I).

To assess whether pharmacological reactivation of PI3K/AKT signaling could modify the reduction in NOX4 expression associated with ENO1 knockdown, Ang II-stimulated si-ENO1 cardiomyocytes were treated with the PI3K-activating phosphopeptide 740Y-P. Under these experimental conditions, 740Y-P increased the p-PI3K/PI3K and p-AKT/AKT ratios and partially increased NOX4 expression in ENO1-silenced cells (Figure 6J,K).

Among the primary atrial cardiomyocytes, both siRNA-mediated ENO1 knockdown and AP-III-a4 treatment reduced PI3K/AKT pathway activation and intracellular NOX4 expression. Pharmacological suppression of PI3K phosphorylation also seemed to reduce NOX4 levels. Conversely, 740Y-P treatment partially reactivated PI3K/AKT signaling and increased NOX4 expression in ENO1-knockdown cells. Collectively, these findings support a role for PI3K/AKT signaling in ENO1-associated changes in NOX4 expression.

### 3.5. AP-III-a4 Treatment Is Associated with Attenuated Atrial Structural Remodeling and Lower Atrial Oxidative-Stress-Associated Signals in Ang II-Infused Mice

Our in vitro findings showed that siRNA-mediated ENO1 silencing attenuated Ang II-induced mitochondrial superoxide accumulation and membrane depolarization, whereas both siRNA-mediated ENO1 silencing and AP-III-a4 treatment reduced the expression of hypertrophic markers. AP-III-a4 treatment was also associated with lower PI3K/AKT phosphorylation and NOX4 expression. We therefore examined whether AP-III-a4 treatment was associated with attenuation of atrial remodeling and oxidative stress in vivo.

**Figure 7:**
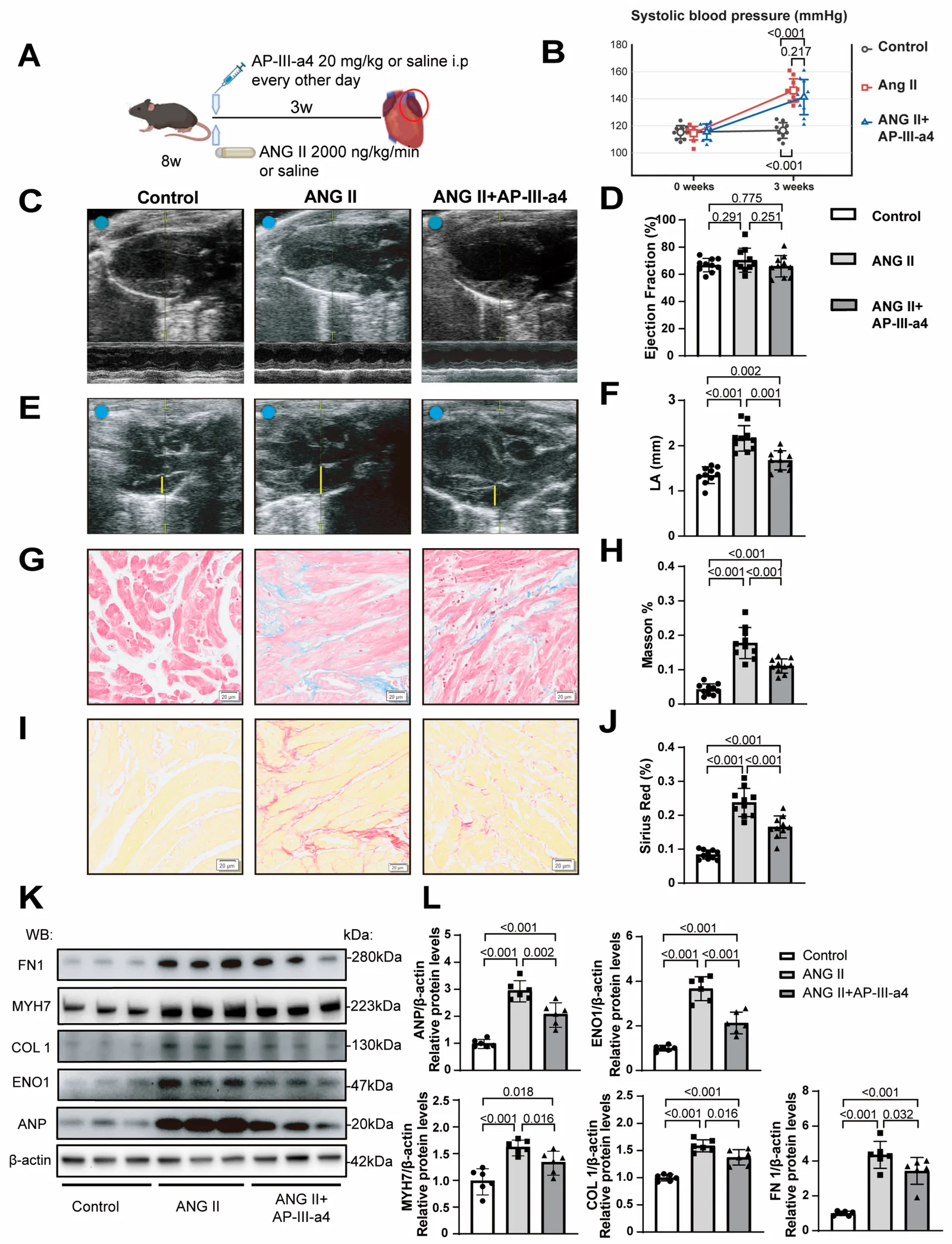
AP-III-a4 treatment attenuates atrial structural remodeling in hypertensive mice. (**A**) Schematic illustration of the in vivo experimental protocol; the red circle identifies the atrial region evaluated. Mice in the control group received a subcutaneously implanted osmotic mini-pump containing saline and intraperitoneal saline injections every other day. Mice in the Ang II group received an Ang II-containing mini-pump and intraperitoneal saline injections every other day. Mice in the Ang II + AP-III-a4 group received an Ang II-containing mini-pump and intraperitoneal injections of AP-III-a4 every other day. (**B**) Statistical results for systolic blood pressure of each group at weeks 0 and 3 (*n* = 10 per group). (**C**,**D**) Echocardiographic assessment of ventricular structure and function (*n* = 10 per group). (**E**,**F**) Echocardiographic assessment and quantification of atrial size; the yellow line indicates the atrial diameter (*n* = 10 per group). (**G**,**H**) Representative Masson’s trichrome staining images and corresponding quantification (*n* = 10 per group). (**I**,**J**) Representative Sirius Red staining images and corresponding quantification (*n* = 10 per group). (**K**,**L**) Western blot analysis and quantification of fibronectin 1 (FN1), collagen type I (COL1), MYH7, ANP, and ENO1 expression in atrial tissues (*n* = 6 per group). Data are presented as mean ± SD. Scale bar: 20 μm.

To further investigate the in vivo effect of AP-III-a4 and its translational potential as a therapeutic target, we then established an Ang II-induced mouse model of hypertension through subcutaneous osmotic mini-pump infusion (Figure 7A). Subsequently, the blood pressure of all the groups of mice was measured. At baseline, systolic blood pressure did not differ among the three groups. Longitudinal analysis showed that the change in systolic blood pressure over time differed significantly among groups (group × time interaction, *p* < 0.001). At week 3, systolic blood pressure was significantly higher in both the Ang II and Ang II + AP-III-a4 groups than in the control group. However, systolic blood pressure did not differ significantly between the Ang II and Ang II + AP-III-a4 groups. Although the mean systolic blood pressure was numerically lower in the AP-III-a4-treated group, this difference was not statistically significant (Figure 7B). Echocardiographic assessment failed to show any major differences among the groups in ventricular function after 3 weeks of treatment, indicating that ventricular remodeling had not yet occurred at this stage (Figure 7C,D). However, at the atrial level, Ang II infusion was associated with larger atrial dimensions, whereas the Ang II + AP-III-a4 group had smaller atrial dimensions than the Ang II group. AP-III-a4 treatment significantly alleviated Ang II-associated atrial enlargement (Figure 7E,F). Masson’s trichrome staining showed that atrial collagen deposition was significantly lower in the AP-III-a4-treated mice than in the Ang II group. Sirius Red staining yielded consistent results (Figure 7G–J). Western blotting showed that ENO1, the cardiomyocyte injury markers ANP and MYH7, and the fibrosis indicators FN1 and COL1 were markedly increased in the Ang II group. Treatment with AP-III-a4 significantly reduced the expression of these proteins in mouse atrial tissue (Figure 7K,L). These results indicate that AP-III-a4 treatment attenuates atrial injury and structural remodeling in Ang II-infused hypertensive mice.

**Figure 8:**
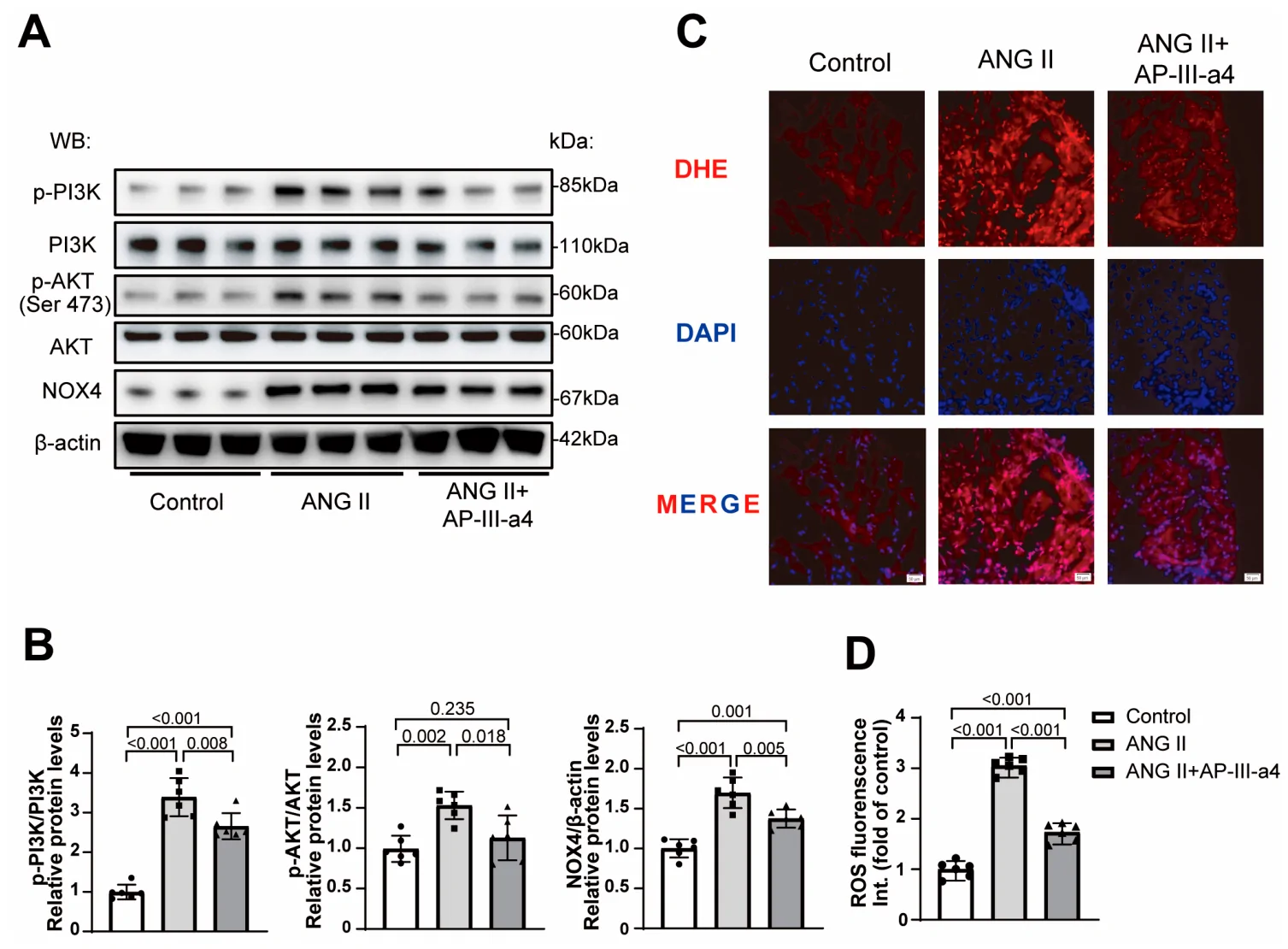
AP-III-a4 treatment was associated with reduced atrial PI3K/AKT phosphorylation and ROS accumulation. (**A**,**B**) Western blot analysis and quantification of AKT, p-AKT, total PI3K, p-PI3K, and NOX4 expression in mouse atrial tissues (*n* = 6 per group). (**C**,**D**) Dihydroethidium (DHE) staining of atrial tissue sections to assess reactive oxygen species (ROS) levels in each mouse group (*n* = 6 per group). Data are presented as mean ± SD. Scale bar: 50 μm.

To examine whether AP-III-a4 treatment in vivo was associated with changes in PI3K/AKT signaling and NOX4 expression consistent with the in vitro observations, we measured the relevant protein levels in atrial tissues. The in vivo results showed that AP-III-a4 treatment significantly reduced PI3K/AKT pathway phosphorylation and NOX4 expression in the atria of Ang II-induced hypertensive mice (Figure 8A,B). To assess the effect of AP-III-a4 treatment on atrial ROS accumulation in vivo, we performed DHE staining of atrial tissues. The result showed that the red fluorescence intensity was significantly lower in the AP-III-a4-treated group than in the Ang II group (Figure 8C,D). These results indicate that AP-III-a4 treatment effectively reduces ROS accumulation in the atria.

Compared with the Ang II group, AP-III-a4-treated mice had smaller atrial dimensions, lower collagen-positive staining, and lower expression of selected hypertrophic and fibrotic markers. In parallel, AP-III-a4 treatment in vivo decreased PI3K/AKT pathway phosphorylation, reduced atrial NOX4 expression, and attenuated ROS accumulation. Together, these findings indicate that AP-III-a4 treatment was associated with concurrent attenuation of atrial structural remodeling and oxidative stress in Ang II-infused mice.

### 3.6. AP-III-a4 Treatment Attenuates AERP Shortening and Shortens Pacing-Induced AF Duration in Hypertensive Mice

**Figure 9:**
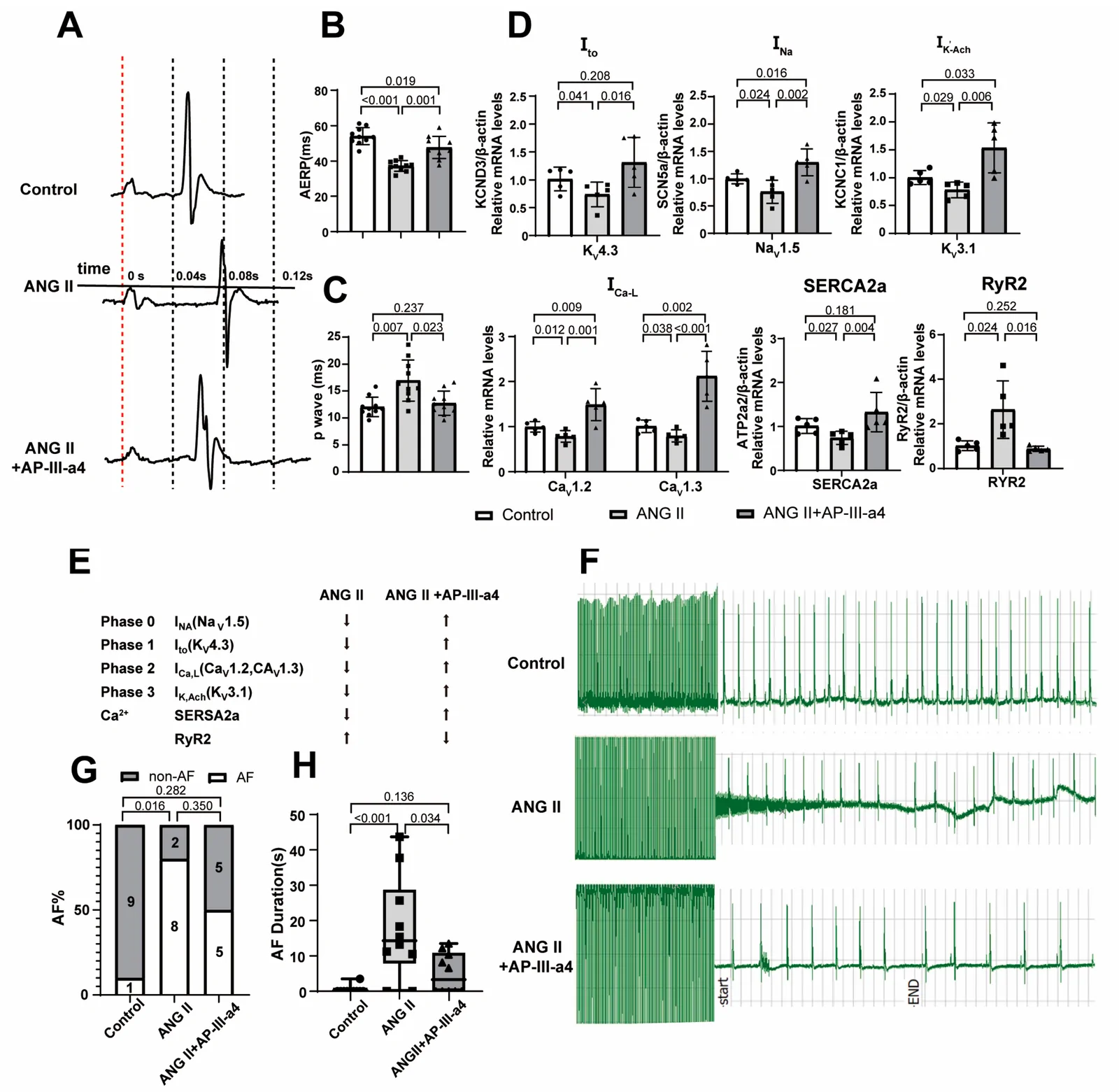
AP-III-a4 treatment attenuated AERP shortening and the duration of AF in Ang II-infused mice. (**A**) Representative surface electrocardiographic (ECG) recordings from mice in each group. The red line indicates the onset of the P wave, defined as 0 s, and the black dashed lines mark 0.04-s intervals. (**B**,**C**) Quantification of AERP and P-wave duration (*n* = 10 per group). (**D**,**E**) RT-qPCR analysis of mRNA expression of atrial action potential-related ion channel genes in each group (*n* = 5 per group). In panel E, downward and upward arrows summarize decreases and increases in transcript abundance, respectively, for Ang II versus control and Ang II + AP-III-a4 versus Ang II. (**F**) Representative surface ECG recordings during programmed stimulation-induced AF; green traces show the pacing and post-pacing recordings, and the labels ‘start’ and ‘END’ indicate the boundaries of the induced episode. (**G**) AF inducibility in each group. Numbers within the bars indicate the number of mice (*n* = 10 per group). AF inducibility was compared using two-sided Fisher’s exact tests with Holm adjustment. (**H**) Duration of AF in each group (*n* = 10 per group). AF duration was calculated as the mean duration of induced AF episodes for each mouse; mice without successful AF induction were assigned a value of 0. Data in panels (**B**–**E**) are presented as mean ± SD, whereas data in panel H are presented as median (IQR). AF duration was analyzed using the Kruskal–Wallis test followed by Dunn’s multiple-comparison test.

In addition to structural remodeling, alterations in atrial electrophysiology, including changes in refractoriness and conduction-related properties, may contribute to the development of AF. Representative surface ECG recordings obtained from mice in each group are shown in Figure 9A. We first assessed the atrial effective refractory period (AERP) in vivo as a direct measure of atrial refractoriness. Compared with the control group, Ang II infusion significantly shortened AERP, whereas AP-III-a4 treatment significantly reversed this Ang II-associated shortening (Figure 9B). P-wave duration is an indirect surface-ECG index of atrial activation and intra-atrial conduction. Ang II infusion significantly prolonged P-wave duration, and AP-III-a4 treatment reduced this prolongation (Figure 9C).

Alterations in atrial P-wave and AERP parameters appear to be mostly attributable to changes in atrial electrical activation, which depend on the properties of cardiomyocyte action potentials. The remodeling of ion-channel-related genes, including potassium and calcium channels, provides an important basis for these action potential alterations. With this in mind, we considered whether AP-III-a4 treatment might help hypertension-induced ion channel transcriptional remodeling. In atrial tissues from Ang II-induced hypertensive mice, mRNA expression of the phase 0 sodium channel Na_V_1.5, the phase 1 potassium channel K_V_4.3, and the phase 2 calcium channels Ca_V_1.2 and Ca_V_1.3 was markedly reduced, indicating substantial electrical remodeling. In addition, SERCA2a levels declined while RYR2 levels rose, consistent with disturbed intracellular calcium handling. The AP-III-a4 treatment attenuated these Ang II-associated transcriptional changes (Figure 9D,E). To further determine the effects of the AP-III-a4 treatment on AF susceptibility in hypertensive mice, we performed programmed stimulation-induced AF testing in each group. AF inducibility was 1/10 in the control group, 8/10 in the Ang II group, and 5/10 in the Ang II + AP-III-a4 group. AP-III-a4 treatment numerically reduced AF inducibility, but this difference was not statistically significant (*p* = 0.350). Treatment significantly shortened the duration of induced AF episodes (Figure 9F–H).

These results indicate that Ang II alters the expression of genes encoding atrial action potential-related ion channels and increases programmed stimulation-induced AF inducibility and duration. AP-III-a4 treatment attenuated ion channel transcriptional remodeling and significantly shortened pacing-induced AF duration, whereas its numerical reduction in AF inducibility did not reach statistical significance.

## 4. Discussion

The present study identifies ENO1-related signaling as a candidate contributor to hypertension-associated atrial remodeling and susceptibility to pacing-induced AF. ENO1 expression was increased in hypertensive rat atria. In primary neonatal atrial cardiomyocytes, siRNA-mediated ENO1 knockdown attenuated Ang II-induced hypertrophic and oxidative-stress-related changes, and AP-III-a4 treatment produced concordant effects. In vivo, AP-III-a4 treatment was associated with reduced atrial enlargement and interstitial fibrosis, attenuation of Ang II-associated AERP shortening, and a shorter duration of pacing-induced AF. The proportion of mice with pacing-induced AF was numerically lower after AP-III-a4 treatment, although this difference did not reach statistical significance. Together, these observations support a working model in which ENO1-related signaling is associated with PI3K/AKT activity, NOX4 expression, and oxidative stress during hypertensive atrial remodeling.

Hypertension is one of the major modifiable risk factors for AF and promotes atrial remodeling through hemodynamic loading, RAAS activation, autonomic dysregulation, inflammation, oxidative stress, and fibrosis [46]. In our study, we found that, although AF inducibility was not significantly increased and major ventricular abnormalities were not yet evident during early hypertension, atrial structural remodeling was already detectable. Atrial enlargement or fibrosis seems to occur prior to major ventricular abnormalities, supporting the concept that the atrium may be an early target of hypertensive injury [47]. Persistent pressure overload may progressively establish an arrhythmogenic substrate characterized by atrial dilation, extracellular matrix deposition, and conduction heterogeneity [48]. The transcriptomic findings further confirm that mitochondrial dysfunction and oxidative stress are central features of hypertensive atrial remodeling. Altered oxidative phosphorylation, respiratory chain, and mitochondrial membrane pathways were evident in both early and established hypertension. Excess ROS seem to disturb calcium homeostasis [49], ion channel function [50], gap junction coupling [51], and mitochondrial integrity [16], as well as fibroblast behavior and collagen production. Thus, mitochondrial injury [52], oxidative stress [53], and atrial electrical and structural remodeling may reinforce one another in a self-perpetuating cycle.

Even though ENO1 is classically recognized as a glycolytic enzyme, it plays roles in stress phenomena and in intracellular signaling [54]. ENO1 has been reported to be increased in the brains of hypertensive animals [17], and we have also noted such persistent elevation in hypertensive atria. In addition, we observed significantly increased ENO1 expression in the left atrial tissue of patients with AF in two independent human transcriptomic datasets, although individual-level hypertension status was unavailable in both datasets and could therefore not be incorporated as a covariate. Among the results reported to date is evidence that lowering ENO1 helps to reduce isoproterenol-induced ventricular hypertrophy [18]. Together with the persistent upregulation of ENO1 in the atria of hypertensive animal models, these findings suggest that ENO1 may represent a potential molecular link between hypertension-associated atrial remodeling. In Ang II-stimulated primary atrial cardiomyocytes, siRNA-mediated ENO1 knockdown and AP-III-a4 treatment were associated with lower PI3K/AKT pathway activity and NOX4 expression. Within ENO1-silenced cardiomyocytes, treatment with the PI3K activator 740Y-P increased PI3K/AKT phosphorylation and NOX4 expression. From a mechanistic perspective, the present findings support a role for PI3K/AKT signaling in ENO1-associated regulation of NOX4 expression. PI3K/AKT signaling has context-dependent functions in the cardiovascular system and can regulate cell growth, survival, metabolism, and pathological remodeling [55]. Here, ENO1 knockdown or AP-III-a4 treatment reduced PI3K/AKT phosphorylation and NOX4 expression. The NOX family is an important intracellular source of hydrogen peroxide and other ROS. In the heart, NOX1, NOX2, and NOX4 are the principal NOX isoforms expressed. Under various pathological conditions, increased NOX expression promotes cardiac ROS accumulation and thereby contributes to cardiac injury and impaired function [42,56]. Recent evidence also indicates that NOX4 expression can drive atrial fibrosis and AF susceptibility through redox-sensitive remodeling [42,57]. From our own findings, it appears that hypertension can promote the expression of ENO1 in atrial cells, increase the phosphorylation of PI3K/AKT pathway, and increase the accumulation of ROS in atrial cells. These molecular and redox-associated changes may contribute to atrial structural remodeling, altered refractoriness, and increased susceptibility to pacing-induced AF. These findings further support a role for PI3K/AKT signaling in ENO1-associated regulation of NOX4 expression, offering a new therapeutic focus for the future treatment of AF.

The pacing-induced AF phenotype observed after AP-III-a4 treatment likely reflects an integrated effect on the atrial substrate rather than an exclusively direct electrophysiological activity. Attenuation of atrial enlargement and interstitial fibrosis may be particularly important because fibrosis disrupts myocardial continuity and promotes conduction heterogeneity [58]. The reduction in collagen deposition may be particularly relevant to the antiarrhythmic effects observed in this study. Alterations in ion channel expression and AERP also contribute to AF development and progression [51]. AP-III-a4 treatment was also associated with attenuation of AERP shortening and transcriptional changes in selected ion-channel- and calcium-handling-related genes. AP-III-a4 significantly shortened pacing-induced AF duration, whereas its numerical effect on AF inducibility was not statistically significant. These findings suggest that modulating ENO1-related signaling may attenuate atrial structural remodeling and partially ameliorate electrophysiology-related changes in experimental hypertension, providing a rationale for further investigation of this pathway as a potential therapeutic strategy for hypertension-associated AF.

Standard antihypertensive treatment is directed mainly toward a reduction in blood pressure [59]; however, the development of hypertension-mediated organ damage may be only partly explained by blood pressure itself. Hypertension is an important contributor to AF development, and previous studies have demonstrated that the extent of atrial remodeling and the risk of AF are associated, at least in part, with the severity of hypertension. In our study, systolic blood pressure was numerically lower in the AP-III-a4-treated group than in the Ang II group, but this difference did not reach statistical significance; systolic blood pressure remained significantly higher than that in the control group. In contrast, atrial fibrosis, atrial enlargement, and other indices of structural remodeling were markedly attenuated following AP-III-a4 treatment (Figure 7C–J). These findings suggest that the beneficial effects of AP-III-a4 on atrial remodeling may involve additional mechanisms associated with modulation of ENO1-related signaling. Persistent hypertension in treated animals does not demonstrate that the improvement in atrial remodeling was independent of hemodynamic changes. Even a modest reduction in pressure load may have contributed to the observed atrial effects, and the present design cannot separate a direct atrial action from effects secondary to altered hemodynamic load. From this perspective, our findings offer a candidate therapeutic target and suggest ways of limiting hypertension-related organ damage, particularly atrial injury. Nevertheless, the results we present here do not establish AP-III-a4 as a clinical therapy. Human diseases are much more complicated than experimental animal models, and the pharmacokinetics, long-term safety, and therapeutic efficacy of AP-III-a4 require further evaluation. AP-III-a4 was initially reported as an enolase-targeting small molecule [40], but it is not selective for ENO1 and its precise mechanism of action remains uncertain. Previous studies have found little or no direct inhibition of enolase catalytic activity [60,61], whereas other reports have suggested that AP-III-a4 may modulate the non-catalytic functions of enolase [21,33]. Therefore, the effects of AP-III-a4 observed in the present study cannot be attributed exclusively to selective ENO1 inhibition. Chlorogenic acid [62], a plant-derived compound, has been reported to inhibit ENO1 activity; however, its potential role in hypertension-associated atrial remodeling requires further validation in both preclinical and clinical studies.

In summary, ENO1-related signaling is a candidate contributor to hypertension-associated atrial remodeling. The cellular data support a role for PI3K/AKT signaling in ENO1-associated changes in NOX4 expression, whereas the in vivo data show that AP-III-a4 treatment was associated with attenuation of atrial remodeling and shorter pacing-induced AF duration. These findings support further investigation of ENO1-related signaling as a potential therapeutic target in hypertension-associated atrial remodeling.

## 5. Limitations

Several limitations of this study should be acknowledged. First, adult in vivo experiments were restricted to male animals, and potential sex-dependent effects were not evaluated. The sex of neonatal cell donors was not determined; therefore, sex-specific cellular responses could not be assessed. Second, differences across species may introduce biological variability, and neonatal and adult rat atrial cardiomyocytes differ substantially in their metabolic profiles and electrophysiological properties. These differences limit direct extrapolation of the findings to the adult human hypertensive atrium. Third, the target specificity of AP-III-a4 remains uncertain, and its in vivo effects cannot be attributed exclusively to ENO1; cardiomyocyte-specific conditional Eno1 knockout models are required for genetic validation. Fourth, the mechanisms by which hypertension increases ENO1 expression and by which ENO1-associated changes influence PI3K/AKT signaling were not directly investigated. Fifth, programmed electrical stimulation assessed pacing-induced AF rather than spontaneous AF, and direct measurements of atrial conduction velocity, conduction heterogeneity, action-potential properties, calcium transients, and ion-channel protein abundance or function were not performed. Finally, the contribution of the numerical reduction in blood pressure to the observed atrial effects cannot be excluded. These limitations should be addressed in future studies.

## 6. Conclusions

This study found that long-term hypertension was associated with progressive atrial remodeling and increased susceptibility to pacing-induced AF in rats. Atrial transcriptomic analyses identified persistent ENO1 upregulation during hypertension. Two external human atrial datasets further provided observational support for an association between ENO1 expression and established AF, although individual-level hypertension status was unavailable in both datasets, precluding assessment of hypertension-specific causality. In primary atrial cardiomyocytes, siRNA-mediated ENO1 knockdown attenuated Ang II-associated hypertrophic and oxidative-stress-related changes. Proteomic analysis and subsequent validation experiments suggested that PI3K/AKT signaling is associated with ENO1-related changes in NOX4 expression in Ang II-stimulated primary atrial cardiomyocytes. Finally, in vivo, AP-III-a4 treatment was associated with reduced atrial hypertrophy and fibrosis, attenuation of Ang II-induced transcriptional changes in selected ion-channel-related genes, and significantly shorter pacing-induced AF duration. AP-III-a4 treatment was also associated with a numerical reduction in AF inducibility, although this difference did not reach statistical significance. Further target-specific genetic studies are required.

## Data Availability

The raw RNA-sequencing reads and processed gene-expression matrices generated in this study have been deposited in the NCBI Gene Expression Omnibus (GEO) under accession numbers GSE346715 (12-week left atrial transcriptomic dataset) and GSE348072 (24-week left atrial transcriptomic dataset). The external validation datasets (GSE41177, GSE115574) are publicly available in the Gene Expression Omnibus (GEO) at https://www.ncbi.nlm.nih.gov/geo/query/acc.cgi?acc=GSE41177 (accessed on 25 July 2025) and https://www.ncbi.nlm.nih.gov/geo/query/acc.cgi?acc=GSE115574 (accessed on 25 July 2025), respectively. The mass spectrometry proteomics data have been deposited to the ProteomeXchange Consortium via the PRIDE partner repository with the dataset identifier PXD085550.

## Supplementary Materials

The following supporting information can be downloaded at https://www.mdpi.com/article/10.3390/cells15211970/s1, Figure S1: The impact of hypertension on the ventricles; Figure S2: Hypertension promotes atrial remodeling and facilitates the occurrence of atrial fibrillation; Figure S3: Expression of ENO1 in fibroblasts and ventricles; Figure S4: Validation of si-ENO1 knockdown efficiency. Table S1: Primer sequence used for RT-qPCR.

## Abbreviations

The following abbreviations are used in this manuscript:

AF	Atrial Fibrillation
ENO1	Alpha-Enolase
Ang II	Angiotensin II
ROS	Reactive Oxygen Species
NOX4	NADPH Oxidase 4
PI3K	Phosphatidylinositol 3-kinase
AKT	Protein Kinase B
SHR	Spontaneously Hypertensive Rats
WKY	Wistar–Kyoto
DEGs	Differentially Expressed Genes
DHE	Dihydroethidium
ANP	Atrial Natriuretic Peptide
MYH7	Myosin Heavy Chain 7
FN1	Fibronectin
COL1	Collagen 1
