# Supplementary material for: ENO1-Related Signaling in Hypertension-Associated Atrial Remodeling: Evidence Implicating PI3K/AKT and NOX4
Source: Cells. 2026 Oct 29;15(21):1970. doi: 10.3390/cells15211970 (PMC13762153; doi:10.3390/cells15211970)
Supplement: Supplementary file 1 [file cells-15-01970-s001.zip › cells-4555160-supplementary.pdf]

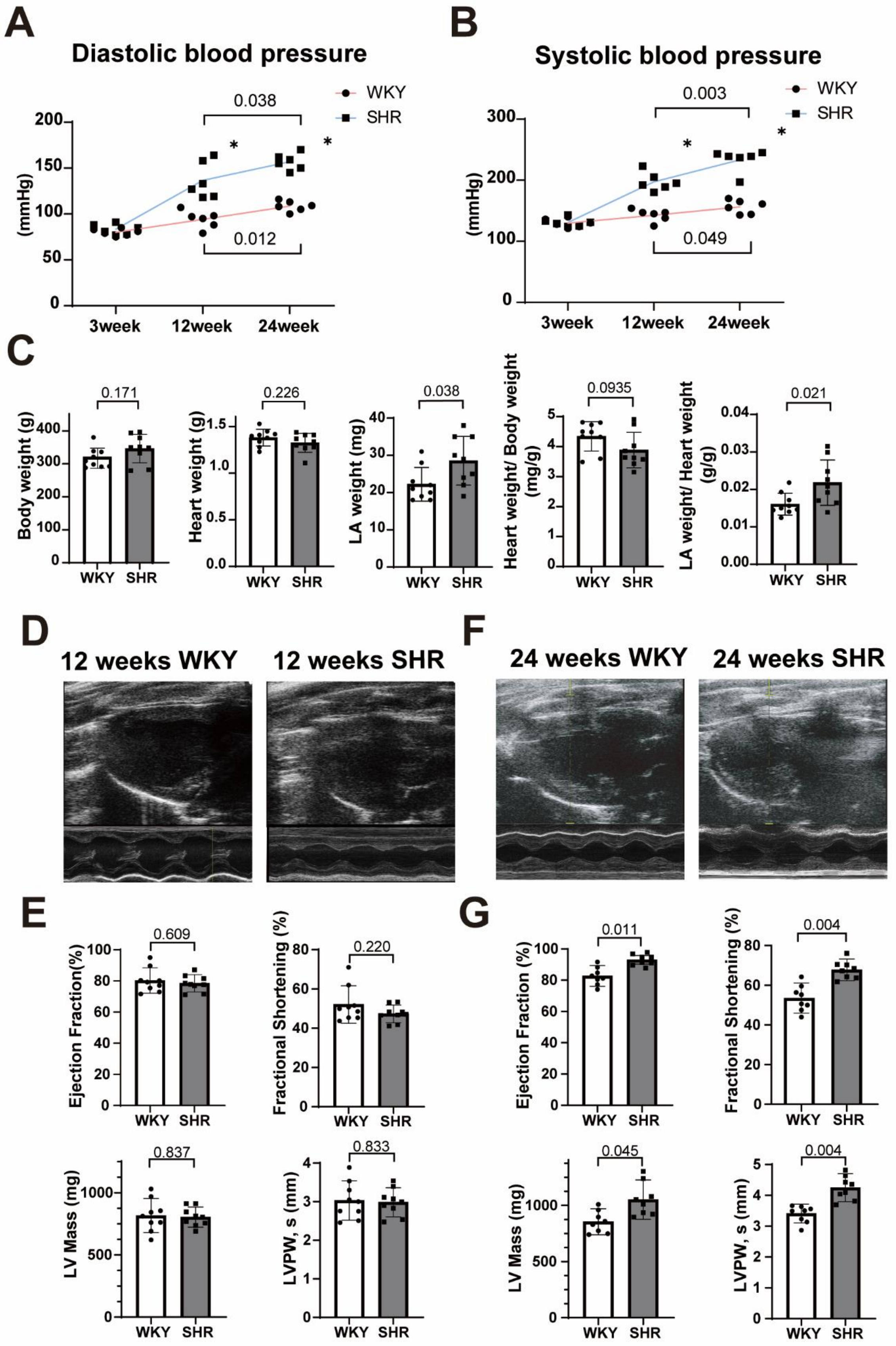
A
Diastolic blood pressure
WKY
SHR
0.038
0.012
(mmHg)
3week
12week
24week
B
Systolic blood pressure
0.003
0.049
C
0.171
Body weight (g)
0.226
Heart weight (g)
0.038
LA weight (mg)
0.0935
Heart weight/ Body weight (mg/g)
0.021
LA weight/ Heart weight (g/g)
D
12 weeks WKY
12 weeks SHR
F
24 weeks WKY
24 weeks SHR
E
0.609
Ejection Fraction(%)
0.220
Fractional Shortening (%)
0.837
LV Mass (mg)
0.833
LVPW, s (mm)
G
0.011
Ejection Fraction (%)
0.004
Fractional Shortening (%)
0.045
LV Mass (mg)
0.004
LVPW, s (mm)

Figure S1 The impact of hypertension on the ventricles

(A,B) Changes in diastolic and systolic blood pressure in spontaneously hypertensive rats (SHR) and Wistar–Kyoto (WKY) rats at 3, 12 and 24 weeks of age (n = 6). *$P<0.05$ for SHR versus WKY rats at the same time point.(C) Body weight, heart weight, left atrial weight, heart weight/body weight ratio, and left atrial weight/heart weight ratio in 24-week-old rats (n = 8).(D,E) Representative 12-week-old SHR and WKY rats echocardiographic images of the ventricles, measureing ejection fraction、fractional shortening, LV mass, LVPW.(n = 9).(F,G) Representative 24-week-old SHR and WKY rats echocardiographic images of the ventricles, measureing ejection fraction、fractional shortening, LV mass, LVPW.(n = 8). Data are presented as mean ± SD.

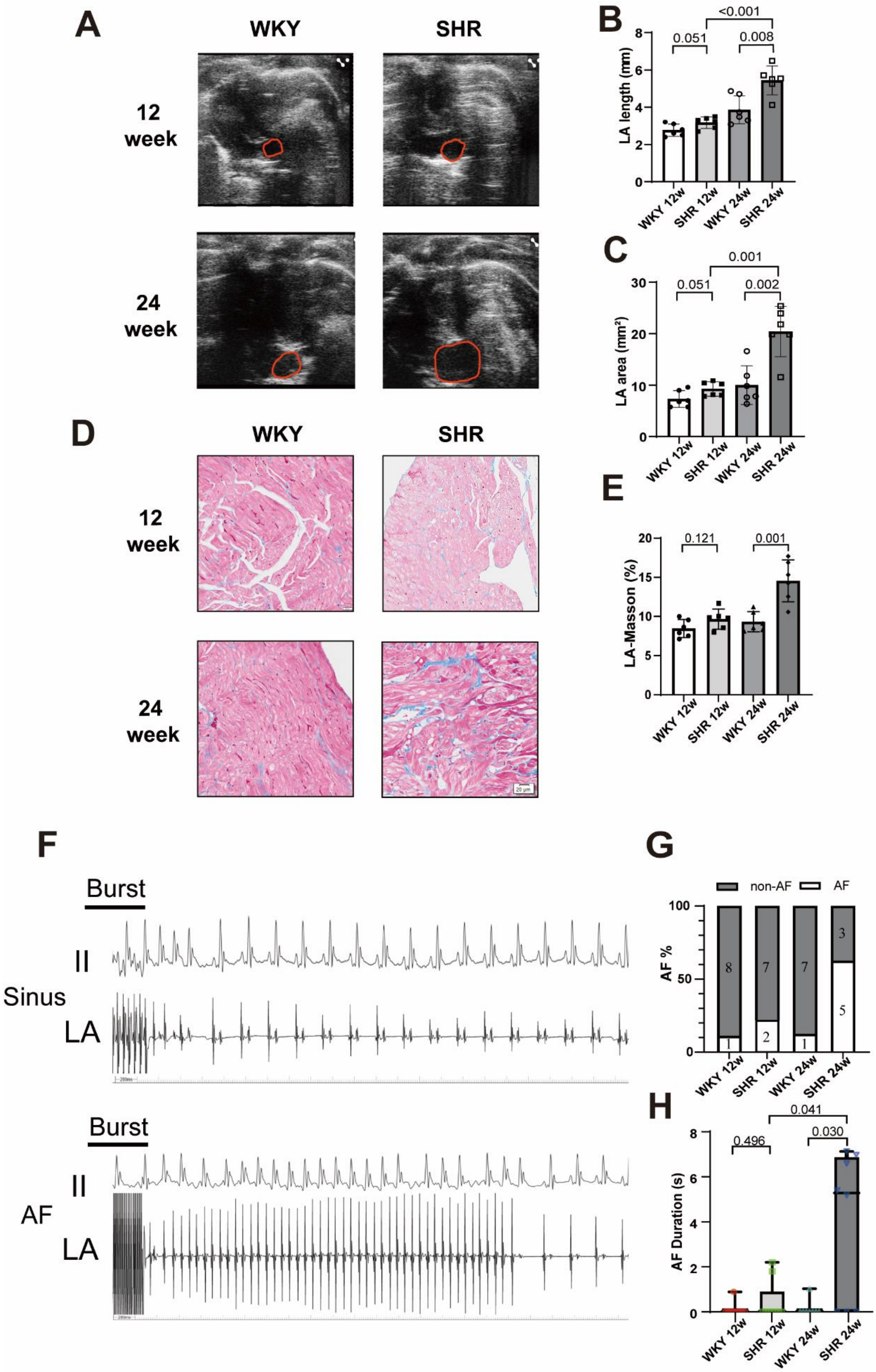
A
WKY
SHR
12 week
24 week
B
LA length (mm)
0.051
<0.001
0.008
WKY 12w
SHR 12w
WKY 24w
SHR 24w
C
LA area (mm²)
0.051
0.001
0.002
D
WKY
SHR
12 week
24 week
E
LA-Masson (%)
0.121
0.001
F
Burst
II
Sinus
LA
Burst
II
AF
LA
G
non-AF
AF
AF %
8
7
7
3
1
2
1
5
H
AF Duration (s)
0.496
0.041
0.030

Figure S2 Hypertension promotes atrial remodeling and facilitates the occurrence of atrial fibrillation

(A)Representative four-chamber echocardiographic images of the atria in 12- and 24-week-old rats. Atrial height was defined as the distance from the mitral valve plane to the roof of the left atrium. The red circle indicates the atrium. (B,C) Quantification of left atrial height and area, respectively (*n* = 6). (D,E) Representative Masson's trichrome staining images of atrial tissues from each group (*n* = 6). Scale bar, 20 μm. (F) Representative surface lead-II ECG and intracardiac left-atrial electrograms following burst pacing, showing unsuccessful and successful AF induction. (G) AF inducibility in the different groups of rats, numbers within the bars indicate the number of rats. AF inducibility was compared using two-sided Fisher's exact tests with Holm adjustment. (*n* = 8-9). (H) AF duration in the different groups of rats (*n* = 8-9). AF duration was calculated as the mean duration of induced AF episodes for each rat; rats without successful AF induction were assigned a value of 0. Data in panels **B**,**C**,**E** are presented as mean ± SD, whereas data in panel H are presented as median (IQR). AF duration was analyzed using the Kruskal–Wallis test followed by Dunn's multiple-comparison test.

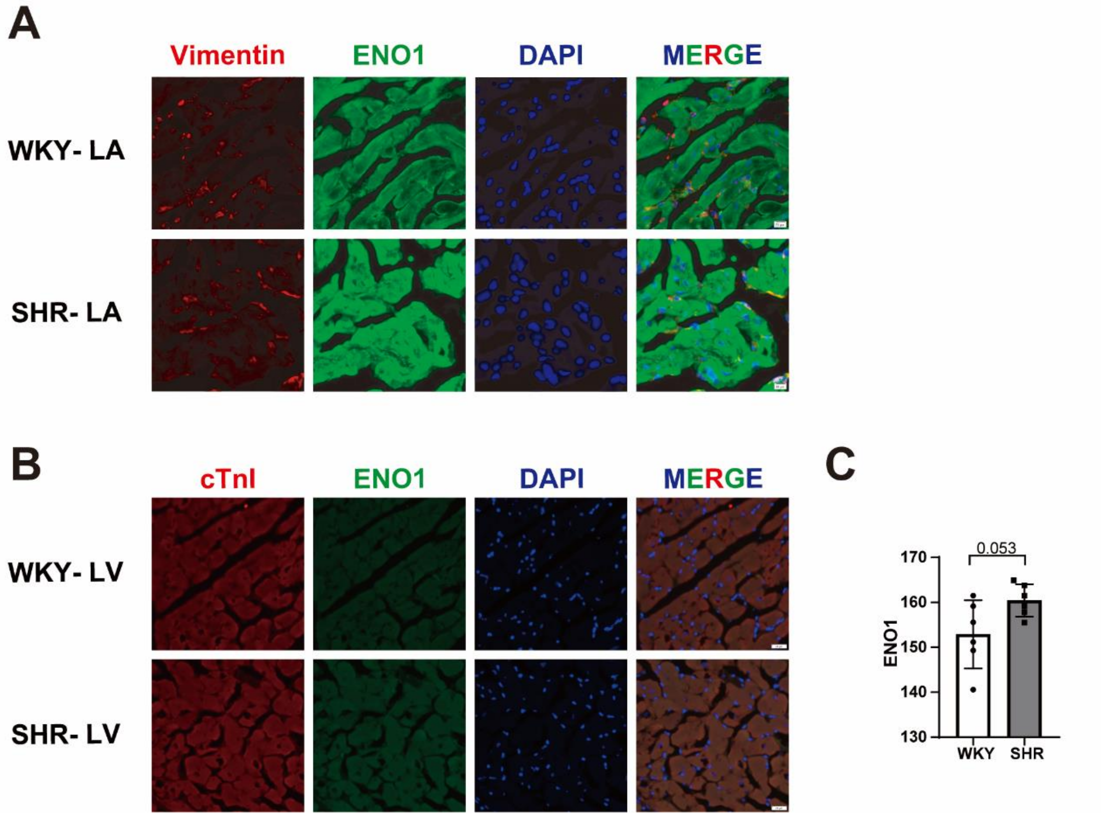

Figure S3 Expression of ENO1 in fibroblasts and ventricles

(A) Representative atrial sections stained for vimentin (red), ENO1 (green), and nuclei (DAPI, blue). Vimentin was used as a fibroblast-associated marker. (B) Representative ventricular sections stained for cTnI (red), ENO1 (green), and DAPI (blue). (C) Quantification of ventricular ENO1 mean fluorescence intensity (n = 6 per group). Data are presented as mean ± SD. Scale bar, 20 μm.

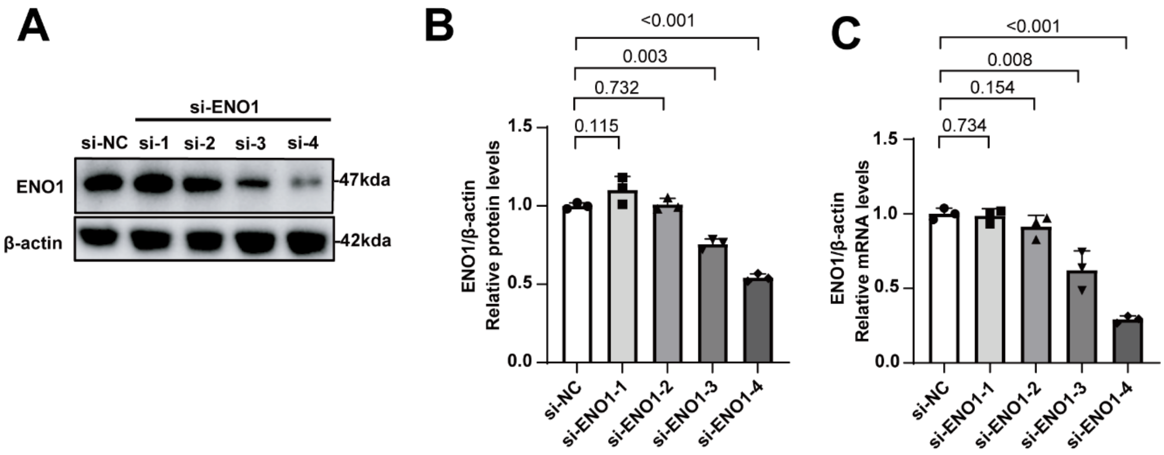

Figure S4 Validation of si-ENO1 knockdown efficiency.

Four siRNAs targeting ENO1 were designed and designated si-ENO1-1 (si-1), si-ENO1-2 (si-2), si-ENO1-3 (si-3), and si-ENO1-4 (si-4). (A) ENO1 knockdown efficiency was evaluated at the protein level for each siRNA. (B) Quantification of knockdown efficiency ($n = 3$). (C) RT-qPCR validation of ENO1 knockdown efficiency ($n = 3$). Based on these results, si-ENO1-4, which showed the highest knockdown efficiency, was selected for subsequent experiments. All si-ENO1 treatments described in the main text refer to si-ENO1-4. Data are presented as mean ± SD.

Gene name	Forward primer (5’-3’)	Reverse primer (5’-3’)
MYH7-rat	CTCAGTCATGGCGGATCGAG	TTCACAGTCACCGTCTTGCC
ANP-rat	GCAAACATCAGATCGTGCCC	GGTGGTCTAGCAGGTTCTTGAAA
BNP-rat	AGCTGCTGGAGCTGATAAGAG	TAAAACAACCTCAGCCCGTCA
ENO1-rat	TACCCTTGCAAGATCAACGG	CATGACCAGCTCAGCAATCT
NOX4-rat	CACCAGCATCCCTTCTCC	CCCGAGGTACAGCTCAAT
β-actin-rat	TGTCACCAACTGGGACGATA	GGGGTGTTGAAGGTCTCAAA
ANP-mouse	GCTTCCAGGCCATATTGGAG	GGGGGCATGACCTCATCTT
ENO1-mouse	TGCGTCCACTGGCATCTAC	CAGAGCAGGCGCAATAGTTTTA
NOX4-mouse	TGCCTGCTCATTTGGCTGT	CCGGCACATAGGTAAAAGGATG
SCN5A-mouse	ATGGCAAACTTCCTGTTACCTC	CCACGGGCTTGTTTTTCAGC
KCNJ3-mouse	GGGGACGATTACCAGGTAGTG	CGCTGCCGTTTCTTCTTGG
KCNC1-mouse	TCCACCACTAATCTCCCTTTCTC	GACTCAGGGGAAAACATCCCA
CACNA1C-mouse	GAAGGCACTGAGAGCGTTCC	GCTCCAGGCCGATAATAGCAT
CACNA1D-mouse	AGAGGACCATGCGAACGAG	CCTTCACCAGAAATAGGGAGTCT
RyR2-mouse	AAAAGTGCGTGTTGGAGATGA	CACCGCCAATGAGATAGCCT
ATP2A2-mouse	GAGAACGCTCACACAAAGACC	CAATTCGTTGGAGCCCCAT
β-actin-mouse	GGCTGTATTCCCCTCCATCG	CCAGTTGGTAACAATGCCATGT

Table S1: Primer sequence used for RT-qPCR.
